# A dynamic structural framework for the allosteric regulation of Hsp70 chaperones

**DOI:** 10.1016/j.jbc.2025.110516

**Published:** 2025-07-24

**Authors:** Lukas Rohland, Roman Kityk, Luka Smalinskaitė-Wolf, Veronika Lashkul, Matthias P. Mayer

**Affiliations:** Center for Molecular Biology of Heidelberg University (ZMBH), DKFZ-ZMBH-Alliance, Heidelberg, Germany

**Keywords:** molecular chaperones, Hsp70, protein folding, allostery, conformational dynamics, allosteric signal transmission

## Abstract

Central to allosteric signaling mechanisms are three processes: (I) initial sensing of the allosterically active ligand, (II) allosteric signal relaying from the ligand binding pocket to the allosterically affected parts of the protein, and (III) stabilization of the allosterically activated state. Hsp70 proteins are ATP-driven molecular chaperones that are essential in many organisms across the tree of life and exert their action using an intricate allosteric mechanism. Although pathways of this allosteric signaling mechanism have been outlined, its spatiotemporal organization is poorly understood. Here, we present the structural and dynamic basis of these allosteric signaling pathways for the prokaryotic model Hsp70 DnaK that rationalizes the action of ATP, several of its analogues, and clients on the allosteric signal transmission. Our data reveal that during the initial sensing of ATP, DnaK tolerates chemical alterations in the α-phosphate group of ATP but not in the γ-phosphate group. Amino acid replacements that interfere with the allosteric regulation of DnaK disrupt the ATP-induced relay of allosteric signal transmission at specific steps by altering the mechanics of allostery or the stability of reaction intermediates, or both. Client binding to a DnaK variant that is unreceptive to the client-sent signals that stimulate DnaK’s ATPase activity, also shows diminished client-induced conformational remodeling of ATP-bound DnaK, suggesting that client-induced conformational changes in DnaK are needed to trigger ATP hydrolysis. Based on these observations, we formulate a dynamic structural framework for the allosteric regulation of Hsp70 chaperones, linking the molecular mechanics of Hsp70s to their biochemical properties.

The 70-kDa heat shock proteins (Hsp70s) are ATP-dependent molecular chaperones and central components of cellular quality control networks found across the tree of life. As such, Hsp70s surveil proteins at all stages of their entire life cycle, assisting polypeptide folding on the ribosome, controlling activity of some proteins in their natively folded state, refolding proteins when denatured under stress conditions, and supporting protein degradation by cellular proteolysis machineries (1). Central to their function is a highly conserved and intricate allosteric mechanism that modulates the affinity of Hsp70 chaperones to their client proteins in a nucleotide-dependent manner (2). When ATP is bound in the N-terminal nucleotide-binding domain (NBD) of Hsp70s, their C-terminal substrate-binding domain (SBD) is mostly in an open conformation. This allows Hsp70s to interact with clients with high on- and off-rates, resulting in low-affinity interaction. Binding of a client, however, stimulates the hydrolysis of ATP, converting Hsp70 into the ADP-bound state where the SBD is mostly in a closed conformation, displaying low on- and off-rates for clients. This effectively increases the affinity to clients by several orders of magnitude (3). The exchange of ADP for ATP restores the low-affinity state and causes the release of a bound client. The continuous interconversion between the ATP- and ADP-bound state drives the Hsp70 activity cycle of binding and release of clients (4). To modulate the affinity of the SBD to clients in response to ATP-binding, the NBD has to recognize ATP and transmit a signal to the SBD through the entire protein, including a highly conserved interdomain linker that is flexible in the presence of ADP (5, 6, 7, 8, 9, 10). Conversely, to stimulate the hydrolysis of ATP by the NBD, the client must transmit a signal from the substrate binding groove to the NBD (11, 12). How these signals can travel from one end of the Hsp70 to the other, and which structural changes are needed to pass on a signal, is not well understood.

Decades of Hsp70 research have uncovered numerous amino acid replacements that disrupt the allosteric signal transmission (Fig. 1*A*), which, together with structural work, led to the proposal of hypothetical pathways through which allosteric signals in Hsp70 chaperones are transmitted (Figs. S1–S3) (12, 13, 14, 15, 16). However, signal transduction in Hsp70 is not a static process like transmission of an electric signal through a wire network but requires an ensemble of conformational changes. To better understand this intramolecular signal transduction process, it is necessary to analyze how individual amino acid replacements alter kinetics of conformational changes and conformational equilibria to interrupt allosteric signaling. We recently developed a methodology that allows the study of conformational dynamics in DnaK with high spatial (5–15 Å) and temporal resolution (down to 1 ms). One key observation we made in this study was that conformational changes following ATP binding in DnaK occur with different rates, suggesting a sequential mechanism for signal transduction starting in the NBD and propagating into the SBD (17). To revisit previous work and test the proposed pathway for the allosteric regulation of Hsp70 chaperones, we made use of our tools to resolve conformational changes in Hsp70 chaperones. We analyzed the conformational dynamics of the prokaryotic model Hsp70 DnaK on binding of several ATP analogs that feature chemically modified phosphate groups and in the background of various amino acid replacements that map onto the proposed allosteric pathway in the absence and presence of clients (Fig. 1*A*) (5, 8, 11, 12, 17, 18, 19, 20). Our results allow us to complement previously postulated pathways of allosteric signaling with time-resolved structural data and propose a structural and dynamic framework for the pathway of allosteric regulation of Hsp70 chaperones.

**Figure 1:**
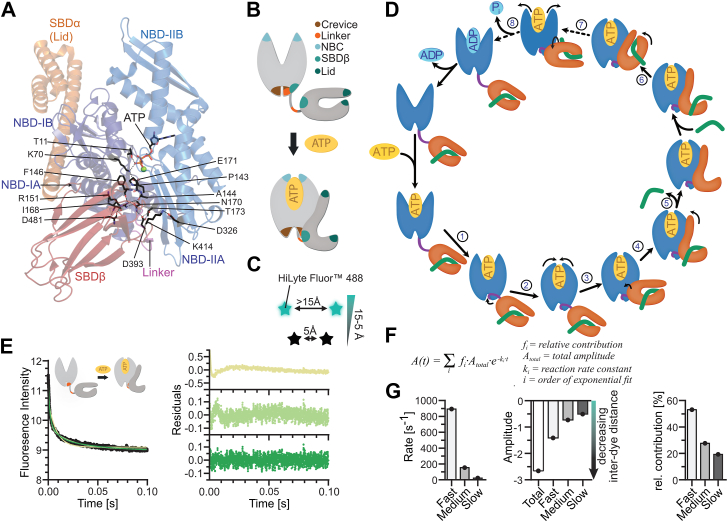
**Proximity-induced quenching of fluorescence to investigate the conformational dynamics of DnaK.***A*, overview over the ATP allosteric signaling pathway. Shown is a cartoon representation of *E. coli* DnaK in the ATP-bound open, domain-docked conformation with NBD lobe I in *dark blue*, NBD lobe II in marine blue, SBDβ in *dark red*, and SBDα in *orange*, and residues that have been demonstrated to be important to process ATP-induced allosteric signals in Hsp70 chaperones as sticks in atom colors with carbon in *black*. *B*, probes used to resolve the conformational dynamics of DnaK. Five pairs of cysteines were placed strategically to monitor allostery-related conformational changes after labeling the probes with HiLyte Fluor 488. *C*, the photoinduced electron transfer (PET) effect underlies the proximity-induced quenching of fluorescence and allows to resolve conformational change between 5–15 Å (21). *D*, conformational changes previously observed on binding of ATP to apoDnaK (Steps 1–5) and on binding of a peptide substrate to DnaK·ATP (Steps 6–8). 1, crevice remodeling and linker docking; 2, NBC closure; 3, SBDβ docking to NBD IB; 4, opening of the α-helical lid; 5, peptide dissociation; 6, lid closure on peptide binding; 7, SBDβ dissociation from NBD IB, partial opening of the NBC, and potentially rotation of the SBDβ; 8, ATP hydrolysis and phosphate release. Of note, kinetic measurements of steps 1–4 were performed in the absence of a bound peptide as peptides influence the starting equilibrium and the total amplitude through rebinding. *E*, exemplary fluorescence traces (*left panel*) recorded for the linker probe on ATP binding to apoDnaK with fits for mono, bi, and tri-exponential functions to the traces (*left panel*) and their residuals (*right panel*). *F*, equation used to fit fluorescent traces. *G*, obtained reaction rate constants (*left*), amplitudes (*middle*), and relative contributions of individual phases to the total amplitude (*right*) from tri-exponential fits (*E*) to fluorescent traces. Note that negative amplitudes indicate a decrease (docking, closing) in the interdye distance and a positive amplitude an increase (undocking, opening).

## Results

### On the interpretation of the kinetic data of conformational changes in DnaK

To obtain time-resolved structural data, we used our recently established fluorescence quenching method (17). Briefly, we labeled DnaK double-cysteine variants with fluorescent dyes that undergo a distance-dependent quenching of fluorescence (Fig. 1, *B* and *C*). At distances below 5 Å, the fluorescence of the dyes is largely quenched (8, 21). Increases in distance relieve the quenching, and above 15 Å, the fluorescence quenching is absent. Thus, our method allows us to resolve relatively small conformational changes between 5 and 15 Å. The pairs of cysteines were introduced at sites that undergo distance changes >5 Å on binding of ATP to nucleotide-free Hsp70 as judged from available crystal structures (Table S1) and thus report on allostery-related conformational changes. These changes include remodeling of the lower crevice between the NBD subdomains IA and IIA, the docking of the linker into the lower crevice of the NBD, the closing of the nucleotide binding cleft (NBC), the docking of the SBDβ onto subdomain IB of the NBD (SBDβ-IB), and the opening of the α-helical lid in the SBD (steps 1–4 in Fig. 1*D*). Our previous fluorescence data also showed that when peptides bind to DnaK·ATP the α-helical lid closes and the SBDβ detaches from subdomain IB prior to ATP hydrolysis and without linker undocking (Steps 6 and 7 in Fig. 1*D*) (17). Crystallographic data also suggest a rotation of the SBDβ by some 180° when peptide is bound to DnaK·ATP (step 7 in Figs. 1*D* and S3) (15). Our fluorescence data were in conflict with such a structure, in particular when a protein substrate was used and in the presence of DnaJ (17).

To obtain kinetic data, we mixed these double-cysteine variants in the apo state with ATP in a stopped-flow device and recorded changes in the fluorescence intensity over time. A sum of exponential functions was fit to the fluorescence traces (Fig. 1, *E* and *F*). Each exponential term of the equation yields two parameters: the rate constant and the amplitude of the change in fluorescence. Rate constants, or rates, are a measure of the time a population of DnaK molecules needs to transit from one conformational equilibrium to another (17). The amplitudes can be understood as a proxy for the change in distance between the fluorophores averaged over the entire population of molecules, and give information about the directionality of a conformational change and the equilibrium conformation of the molecules. If the amplitude is positive, the inter-dye distance increases, and a particular region opens or two structural elements undock from each other. If the amplitude is negative, the inter-dye distance decreases, indicating whether a region closes or two structural elements dock. As observed previously (17), the exponential fit for binding ATP to wild-type DnaK (DnaK_wt_) is tri-phasic and thus yields additional parameters we refer to as relative contribution toward the total amplitude (Fig. 1, *E*–*G*). Our earlier work (17) suggests that the triphasic behavior on ATP binding stems from a preexisting conformational equilibrium of nucleotide-free DnaK (apoDnaK) (for extensive discussion, see supporting information of (17)). Each subpopulation has a different starting conformation and displays a distinct ATP-association constant. To infer mechanistic insights, we compare the rate constants of a reaction in the presence of an amino acid replacement that impacts allostery with a control that contains the wild-type residue. We compare the weighted rate constants, the average of all rate constants weighted by their relative contribution to the total amplitude, to understand the influence of the perturbation on the entire population of molecules. The comparison of the rate constants between DnaK_wt_ and a variant defective in allostery allows inference on the molecular mechanics of the reaction and comparison of the amplitudes of differences in the equilibrium conformation of DnaK molecules that explain observed biochemical properties of a variant, *e.g.,* lowered or increased ATP hydrolysis rates or decreased client release rates. Thus, together, our comparisons deliver a structural dynamic framework for the Hsp70 allostery that connects the dynamics of Hsp70 with its biochemical output.

### Geometry and electrostatics of the γ-phosphate are important for eliciting an allosteric signal

**Figure 2:**
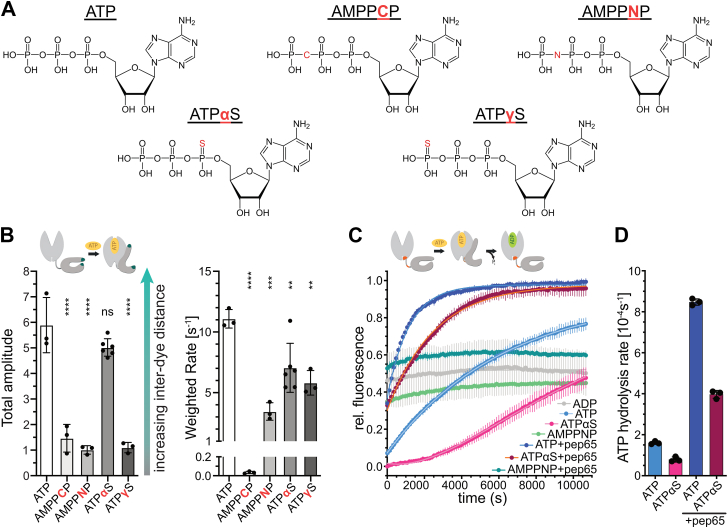
**Conformational dynamics of DnaK on binding of ATP analogs.***A*, overview of the ATP analogs investigated in this study. *B*, total amplitudes (*left panel*) and weighted rates (*right panel*) for binding of ATP and ATP analogs to apoDnaK_lid_ (HiLyte Fluor 488-labelled DnaK_E430C,R547C_). Weighted rates were calculated by weighting the rate of the individual phases according to their contribution towards the total amplitude and are used to characterize the population average. Shown are the mean and standard deviation for at least three independent replicates. Statistical significance of the values was assessed with ordinary one-way ANOVA and Dunnett's multiple comparison; ns, not significant; ∗∗, *p* < 0.01; ∗∗∗, *p* < 0.001; ∗∗∗∗, *p* < 0.0001. *C*, fluorescence signal changes within DnaK_linker_ (HiLyte Fluor 488-labeled DnaK_E217C,L392C_) on binding and hydrolysis of ATP or its analogues (ATPαS, AMPPNP) in the absence or the presence of pep65. *D*, rates of ATP or ATPαS hydrolysis by DnaK_linker_ in the absence or presence of pep65. Shown are the mean and standard deviation for three independent replicates.

To validate our experimental approach and to understand how the chemical properties of the phosphate groups affect the initial sensing of ATP, we compared the ability of ATP and some of its analogs to elicit conformational rearrangements in the SBD. We tested ATP analogs that were modified at the α-phosphate or γ-phosphate (ATPαS and ATPγS), or had replacements in the βγ-phosphoanhydride bond (AMPPCP and AMPPNP) (Fig. 2*A*). To monitor allosteric signal transmission to the SBD, we used the HiLyte Fluor 488-labelled variant DnaK_E430C,R547C_ that reports on the SBD lid opening (here called DnaK_lid_; Fig. 1*D* step 4).

Consistent with previous observations, the total amplitudes for AMPPNP-induced opening of the SBD lid were significantly smaller than for ATP-induced opening (Fig. 2*B*) (22). A related analog, AMPPCP, in which the oxygen of the βγ-phosphoanhydride bond is replaced with a methylene instead of an imido group, induced a comparably small opening. Similarly, ATPγS, in which one of the non-bridging oxygen atoms at the γ-phosphate of ATP was replaced with sulfur, also exhibited a strongly reduced opening of the lid. Solely for the nucleotide analog ATPαS, in which one of the non-bridging oxygen atoms at the α-phosphate group is replaced with sulfur, we observed a nearly complete opening of the lid. Whereas the AMPPNP, ATPαS, and ATPγS-induced changes in fluorescence occurred with weighted average rates of a third to half of the ATP-induced rate, the AMPPCP-induced fluorescence changes displayed significantly lower rates (ca. 1/325^th^; Fig. 2*B*; for rates and amplitudes of fast, medium, and slow phase see Figs. S4–S6, and for discussion of the fast phase subpopulation see Supporting Note 1).

Our observations that ATPαS binding to DnaK opens the lid similar to ATP binding to DnaK and that the AMPPCP, AMPPNP, and ATPγS-induced opening is strongly reduced as compared to ATP-induced opening are consistent with a recently published smFRET study (23).

**Figure 3:**
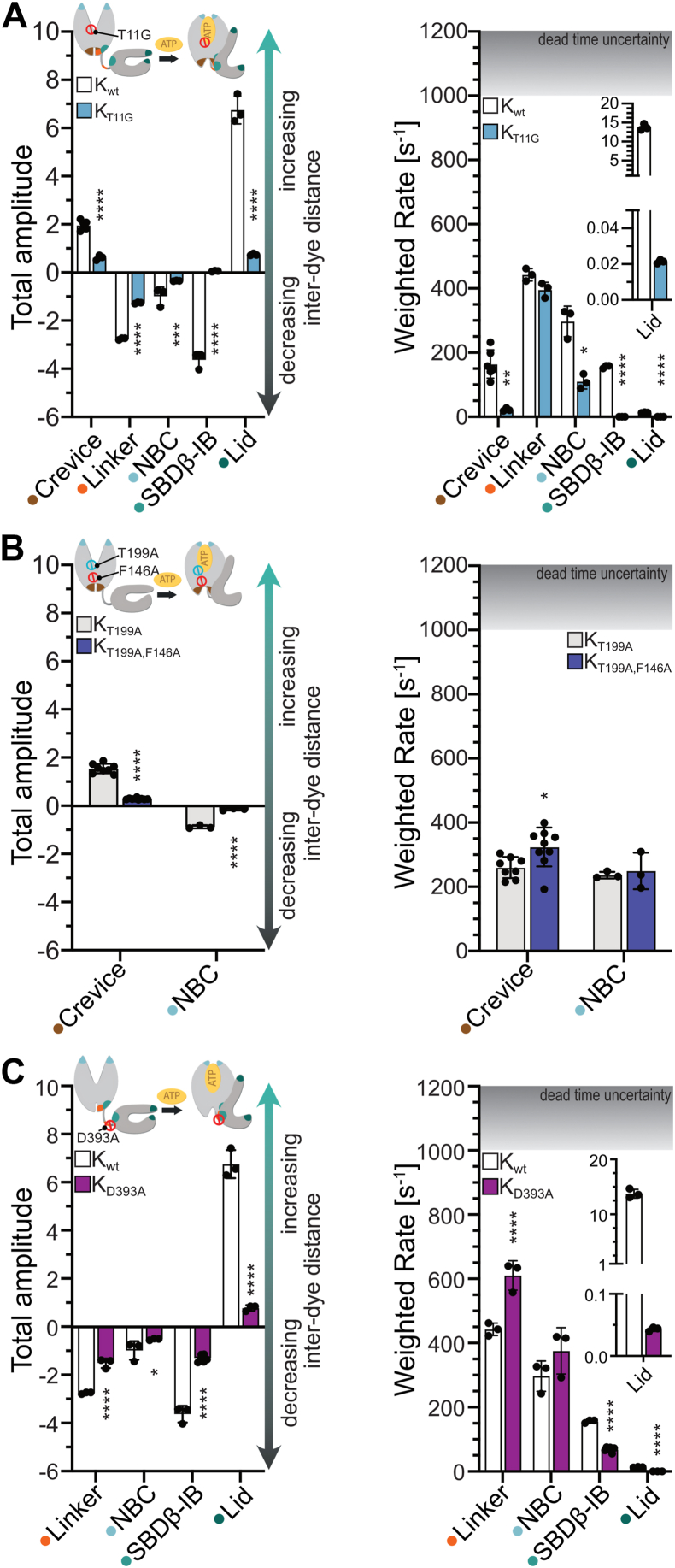
**Conformational dynamics of DnaK variants with amino acid replacements in nucleotide-binding domain or interdomain linker.***A-C*, total amplitudes (*left panel*) and weighted rates (*right panel*) for ATP binding to apoDnaK probes containing T11G in the NBD (*A*), F146A and T199A in the NBD (*B*), and D393A in the interdomain linker (Fig. S1) (*C*). As a reference for all experiments, amplitudes and rates of ATP-induced fluorescence changes in DnaK_wt_ (A + C) or DnaK_T199A_ (*B*) are shown. Note: the shaded area indicates the increased uncertainty of rates larger than 1000 s^−1^ due to the dead time of the stopped-flow instrument. Shown are the mean and standard deviation for at least three independent replicates. Statistical significance was assessed using *t* tests for the crevice data and ordinary one-way ANOVA with Šídák’s multiple comparison for all other comparisons (see explanation on statistical significance assessment in Experimental Procedures); ∗, *p* < 0.05; ∗∗, *p* < 0.01; ∗∗∗, *p* < 0.001; ∗∗∗∗, *p* < 0.0001; no indications, differences are not significant. Insets show zooming into data.

As the phosphoanhydride bond between β and γ phosphate is intact in ATPαS and residues that are implicated in ATP hydrolysis are in contact with the γ phosphate (Fig. 6*B*, (8)), we wondered whether Hsp70s can hydrolyze ATPαS. To measure ATPαS hydrolysis, we took advantage of the previously characterized HiLyte Fluor 488-labelled DnaK_E217C,L392C_ (DnaK_linker_) (17). On ATP binding, this variant exhibits a strong reduction of fluorescence and subsequent ATP hydrolysis should lead to an increase in fluorescence. Furthermore, peptide binding to ATP-bound DnaK_linker_ does not lead to a prominent change in fluorescence, and thus this variant could be used to monitor whether peptide substrates stimulate the hydrolysis of ATPαS. To establish this assay as a general non-radioactive single turnover-type assay, we mixed equal concentrations of DnaK_linker_ and ATP, ATPαS, ATP + pep65 (σ^32^-_195_MAPVLYLQDKSSN_207_, encompassing the binding site of DnaK in the *Escherichia coli* heat shock transcription factor σ^32^; (24)) or ATPαS + pep65. As controls, we included reactions in which DnaK_linker_ was mixed with AMPPNP, AMPPNP + pep65, or ADP. We monitored the change in fluorescence using 384-well microtiter plates in a plate reader at 22 °C (Fig. 2*C*). Of note, we would like to point out that this type of single turnover assay does not allow to monitor the J-domain protein mediated stimulation of Hsp70 ATP hydrolysis, because the fluorophores are incompatible with J-domain binding (See (17) supporting information). For all control reactions (ADP, AMPPNP, AMPPNP + pep65), we observed very small changes in fluorescence. For the reactions with ATP, ATP + pep65, and ATPαS + pep65, we observed an initial steep increase in fluorescence that leveled off, following a single exponential function as expected for a single turnover ATP hydrolysis reaction. The initial drop of fluorescence caused by binding of ATP and ATPαS, respectively, apparently occurred within the dead time of the setup (τ = 2.3 ms, weighted average from Fig. 3*A*). For the reaction that contained ATPαS, fluorescence changed with biphasic kinetics. In the first phase, fluorescence increased very slowly and, in the second phase, continued into a single-exponential saturation curve. The second phase clearly indicated that DnaK can hydrolyze ATPαS. We hypothesize that on binding of ATPαS, DnaK transits slower into the state committed to hydrolysis than when ATP associates. This transition seems to be accelerated by the presence of a substrate peptide, as we did not observe such a first phase in the presence of ATPαS + pep65. The fluorescence data in the presence of ATPαS could be fitted to the equation

$$F=F_{0}+\left(F_{\max}-F_{0}\right)\cdot e^{-k_{1}\left(t-t_{0}\right)}+\left(F_{\max}-F_{0}\right)\left(1-e^{-k_{2}\left(t-t_{0}\right)}\right)$$

with *F*_*0*_ being the quenched, minimal fluorescence after association of ATPαS and *F*_*max*_ the maximal fluorescence at the end of the reaction; *k*_*1*_ encompasses the association rate for ATPαS, but also the dissociation and reassociation before commitment to catalysis, and *k*_*2*_ represents the hydrolysis rate; *t*_*o*_ represents the unobservable dead time. Only *F*_*max*_ and the hydrolysis rate *k*_*2*_ could be determined with high confidence due to the unobserved fluorescence changes during the dead time of the experiment.

In this plate reader assay, ATP was hydrolyzed at a rate of (1.605 ± 0.044)·10^−4^ s^−1^ at 22 °C, which is approximately one fourth of the rate observed in traditional radioactive single turnover experiments at 30 °C (Fig. 2*E*). ATPαS was hydrolyzed with approximately half the rate (0.794 ± 0.073)·10^−4^ s^−1^. The peptide substrate stimulated the rate of hydrolysis of ATP as well as ATPαS ca. fivefold to (8.479 ± 0.091)·10^−4^ s^−1^ (ATP) and (3.971 ± 0.112)·10^−4^ s^−1^ (ATPαS). These data demonstrate that DnaK hydrolyzes ATPαS, and this hydrolysis is stimulated by substrates.

Collectively, these data demonstrate that for the prokaryotic Hsp70 DnaK geometry and electrophilicity of the γ-phosphate are critical for triggering the allosteric signaling mechanics that result in the opening of the lid and release of a bound substrate, consistent with equilibrium measurements by Voith von Voithenberg and colleagues (23). However, this may not be the case for all Hsp70s, as at least the ER-localized Hsp70 HSPA5/BiP seems to be less restrictive about the γ-phosphate configuration. HSPA5 can assume an ATP-like conformation in the presence of ATPγS and AMPPNP (23), although it is currently unknown whether ATPγS and AMPPNP-induced conformational changes in HSPA5 occur at similar rates as ATP-induced changes. Importantly, our work shows that ATPαS induces ATP-like conformational changes with significantly lower rates on the population average and that it is hydrolyzed by DnaK and that hydrolysis is stimulated by clients. Thus, caution needs to be exerted when using ATP analogs, and analogs should always be tested for their ability to mimic ATP in equilibrium and kinetic terms and their susceptibility to catalyzed hydrolysis.

### Nucleotide-binding cleft mutations affect allosteric signal transmission through altered domain dynamics

Having established the requirements for ATP to successfully initiate allosteric signal transmission in our experimental system, we investigated residues of the allosteric signaling pathway in the NBD next (Fig. 1*A* and S1). Our previous work suggested that conformational changes following binding of ATP propagate in a wave-like manner from the NBD to the SBD (see Fig. 1*D*). We hypothesized, in analogy to how signaling cascades are traditionally studied in cell biology, that disruptions to any part of a signaling relay mechanism will inevitably hamper downstream signal transmission, if these parts are constituents of the same pathway, but may not necessarily affect upstream signal transmission. The allosteric signaling pathway within the NBD can be divided into an early part made up of residues that directly contact ATP and a relay that transduces signals to the interdomain interface (18, 20). Thus, we investigated one mutant for each part of the pathway to understand how these perturbations affect the ATP-induced conformational changes (12, 18). To investigate the early stages of ATP-sensing, we chose the T11G amino acid replacement (*E. coli* DnaK numbering used throughout the manuscript except, where stated otherwise; for biochemical properties of mutant Hsp70s see supporting Table S2). This amino acid replacement was originally identified by Wei *et al.* in HSPA5 (BiP/Grp78; T37G) as a variant that had a reduced basal ATPase activity, which could not be stimulated by a substrate peptide. ATP binding to this mutant did not reduce the affinity for a peptide substrate and did not induce a conformational change as determined by partial proteolysis (18). Subsequent work by McKay and colleagues in bovine Hsc70 confirmed the reduced basal ATPase activity and the defect in ATP-induced conformational change as determined by small angle x-ray scattering and tryptophane fluorescence (25).

To investigate the relay part of the allosteric signaling pathway, we used the F146A replacement, previously characterized biochemically in our lab (12). The DnaK_F146A_ exhibits a 40-fold increased ATP association rate but an ATP-induced substrate release rate that was only 1/34^th^ of the wild-type rate. Interestingly, DnaK_F146A_ was proficient in substrate-stimulated ATPase activity and in synergistic stimulation of the ATPase activity by substrate and DnaJ, suggesting divergent pathways of allostery (12). DnaK_F146A_ is defective in refolding denatured luciferase and poorly complements the temperature sensitivity phenotype of a *ΔdnaK E. coli* strain, in contrast to variants that are deficient in substrate-triggered ATP hydrolysis but proficient in ATP-stimulated substrate release (12). F146 forms hydrophobic contacts with P143, a residue we previously showed to act as a switch that stabilizes the ATP-bound state of Hsp70s and is needed to relay allosteric signals from ATP to the NBD-SBD interdomain interface *via* a loop containing V142, P143, A144, Y145, and F146. This loop makes two hydrogen bonds with the interface residue R151 *via* the backbone carbonyls of A144 and F146 (Fig. S1*B*, Movie S1) (20).

For DnaK_T11G_, ATP-induced fluorescence changes in all double-cysteine variants occurred with significantly reduced amplitudes in the total population average, indicating that DnaK does not stably adopt the ATP-bound state (Fig. 3*A*), consistent with the observations by Wei *et al.* for HSPA5 (18) and with small-angle X-ray scattering and tryptophan fluorescence data for bovine Hsc70 (25). Furthermore, this notion is supported by biochemical experiments demonstrating that DnaK_T11G_ released a model client in response to ATP binding with two orders of magnitude lower rates (Fig. S7). Moreover, in the total population average, the kinetics for all ATP-induced conformational changes were slowed down except for linker docking, suggesting defects in the mechanics of signal propagation. Previously, we were unable to dissect the temporal order of ATP-induced crevice remodeling and linker docking (Step 1 in Fig. 1*D*) due to limitations of our experimental setup (17). If crevice remodeling were a requirement for docking of the linker, we would expect to see a drastic slowdown for linker docking in DnaK_T11G_ as well. Since this is not the case, we conclude that linker docking precedes remodeling of the lower crevice, and the remodeling of the lower crevice may represent a conformational change that stabilizes the linker in the docked conformation.

DnaK_F146A_ hydrolyzes ATP at elevated rates (5-fold higher than DnaK_wt_) (12). To ensure that ATP-hydrolysis does not interfere with our kinetic measurements by causing additional conformational changes, we introduced the T199A amino acid replacement in this DnaK variant (26). We and others have previously shown that the T199A replacement does not interfere with the allosteric proficiency of DnaK and that the kinetics of ATP-induced conformational changes are indistinguishable from DnaK_wt_ (17, 26). In DnaK_T199A,F146A_, ATP binding did not induce complete crevice remodeling and NBC closure (steps 1 and 2 in Fig. 1*D*) in the population average (Fig. 3*B*), suggesting that DnaK is unable to stably adopt the ATP-bound state, consistent with biochemical data showing that ATP-induced client-release rates are strongly reduced in this variant as compared to DnaK_wt_ (12). The weighted rate for crevice remodeling is marginally increased for DnaK_T199A,F146A_ as compared to DnaK_T199A_, and the rate for NBC closure is indistinguishable from DnaK_T199A_, suggesting that in principle the mechanics of the NBD is intact. Together, these observations are in line with the previously proposed role of F146 in stabilizing the ATP-bound state of Hsp70 *via* interactions with the P143-switch, which is needed to relay signals to the interdomain interface (12). Whether such a defective ATP-bound state affects client trapping, is not clear, although clients stimulate the already elevated ATPase activity of DnaK_F146A_ further and in synergism with DnaJ (12). However, an inefficient client trapping could explain the inability of this variant to refold luciferase and its poor complementation of the temperature sensitivity phenotype of a Δ*dnaK E. coli* strain.

The observations for DnaK_T11G_ and DnaK_T199A,F146A_ together suggest that interference with ATP sensing (DnaK_T11G_) or the allosteric signal processing in the NBD (DnaK_T199A,F146A_) impact the early conformational changes DnaK needs to completely adopt the final ATP-bound state. Failure to do so, leads to an ADP-like conformation that is incapable of releasing clients at elevated rates (DnaK_T11G_ and DnaK_T199A,F146A_).

### A linker mutant fails to relay signals across the NBD-SBD interdomain interface

Our lab previously demonstrated that the D393A replacement in the interdomain linker renders DnaK unable to adopt the ATP-bound state and to release peptides at elevated rates in response to ATP binding (Table S2). In addition, D393A moderately increases the basal ATP hydrolysis rate, which led us, and other groups, to conclude that the interdomain linker is essential to communicate signals from the NBD across the interdomain boundary to the SBD (5, 6). Furthermore, DnaK_D393A_ does not complement the temperature sensitivity phenotype of a *ΔdnaK E. coli* strain (5). D393 is highly conserved within the entire Hsp70 family and in the ATP-bound state contacts N170 and T173 *via* hydrogen bonds (Figs. S1*B* and S2*B*, Movie S1) and thus has been proposed to be important to stabilize the docking of the SBD onto the NBD in the presence of ATP by decreasing the distance between SBD and NBD (Fig. S2*C*) (17). Therefore, we investigated the consequences of the D393A replacement for the conformational dynamics of Hsp70 and tested whether D393A affects more the kinetics of linker docking, as could be expected of abrogated polar interactions, or the equilibrium of linker docking and to what extent the subsequent NBC closure, SBDβ docking, and lid opening are affected.

Consistent with our previous observations, our data show that DnaK_D393A_ is unable to adopt an ATP-bound state as indicated by a reduction of all amplitudes for all monitored conformational changes (Fig. 3*C*). Moreover, for the total population average, we observed significantly lower rates for SBDβ-IB docking and lid opening (steps 3 and 4 in Fig. 1*D*), but increased docking rates for the linker. Solely the NBC closure rate was similar to what we observed for DnaK_wt_, suggesting that the mechanics of the allosteric signal processing in the NBD (Steps 1 and 2 in Fig. 1*D*) are intact, but deteriorate as the signal crosses the interdomain boundary through the linker, presumably because the linker cannot be kept stably docked in the lower crevice. Importantly, the lowered amplitude for SBDβ-IB docking for DnaK_D393A_ suggests that, on average, the SBD is much further away from the NBD, or a smaller subpopulation is in a docked state, when compared to DnaK_wt_, arguing that the linker promotes docking of the SBD onto the NBD by bringing both domains in close proximity.

With these data, we obtained direct structural evidence that docking of the interdomain linker in Hsp70 is essential for the transmission of signals across the interdomain boundary.

### Docking of the Hsp70 SBD onto the NBD is a prerequisite for opening of the lid domain

Among the earliest interdomain interface residues identified as critical for the allosteric mechanism of Hsp70s are K414 and R151 (Fig. S1) (19, 20). Both, DnaK_K414I_ and DnaK_R151A_ are defective in ATP-induced peptide release, ATP-induced change in tryptophane fluorescence, and peptide-stimulation of the ATPase activity (Table S2), and both have reduced chaperone activity as evidenced by plaque formation of bacteriophage λ (DnaK_K414I_) or refolding of denatured firefly luciferase and complementation of the temperature sensitivity phenotype of a *ΔdnaK E. coli* strain (DnaK_R151A_) (19, 20). The crystal structure of DnaK in the ATP-bound state (8) revealed that the central part of the allosteric signaling pathway in the NBD converges on R151 (Fig. S1), which contacts the peptide backbone of D481 in the SBD *via* a hydrogen bond in the ATP-bound state (8, 9). K414 is a constituent of the SBD part of the allosteric signaling pathway and forms a salt bridge with D326 of the NBD. The SBDβ has been proposed to form a clamp contacting NBD subdomain IIA through K414 and NBD subdomain IA through D481 to arrest the NBD lobes in a rotated conformation (Fig. S1) (12, 13, 14). DnaK_D481A_ has an 80-fold increased basal ATPase rate and is defective in allostery as the ATPase activity is not stimulated by substrate and DnaJ, tryptophane fluorescence is not influenced by ATP, and peptide dissociation is not accelerated by ATP (Table S2). *In vitro* refolding of denatured luciferase is strongly reduced for DnaK_D481A_, but not as much as for DnaK_F146A_, and the variant does not complement the temperature sensitivity phenotype of a *ΔdnaK E. coli* strain (12). Although D481 seems to be crucial for stabilizing the SBD-NBD domain interface, asparagine is more frequently found in this position (96% of all eukaryotic cytosolic Hsp70s and 85% of all DnaK homologs in prokaryotic organisms). In this particular case, D481 forms a hydrogen bond with the peptide backbone amide of I168 and replacement with asparagine should only mildly impact the function of the protein because the carbonyl of asparagine, in principle, should be able to interact with the backbone amide of I168 *via* hydrogen bonds as well, though potentially with a different binding enthalpy, consistent with an altered interdomain interface equilibrium for DnaK_D481N_ as shown previously (11). To understand how amino acid replacements in the interdomain interface affect the conformational dynamics of DnaK, we investigated DnaK_R151A_, DnaK_K414I_, DnaK_D481A_, and DnaK_D481N_ variants.

**Figure 4:**
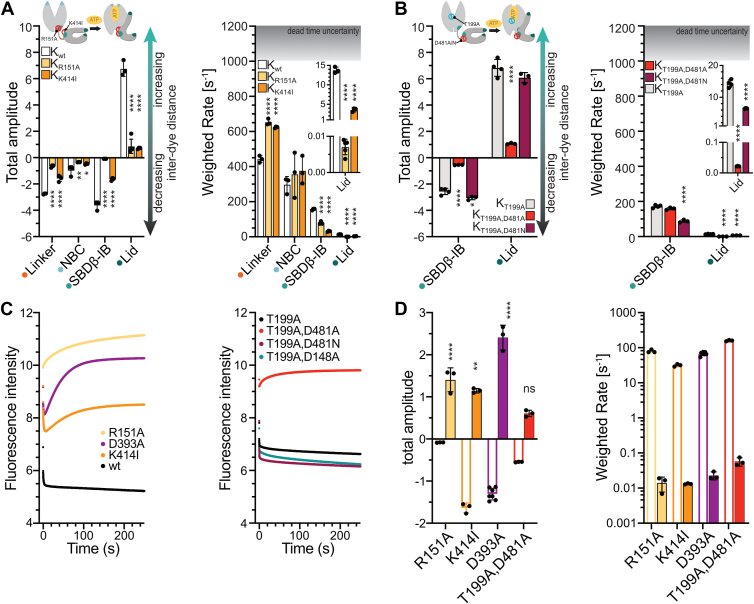
**Conformational dynamics of DnaK variants with amino acid replacements in the NBD–SBD interface.***A-B*, total amplitudes (*left panel*) and weighted rates (*right panel*) for ATP binding to apoDnaK probes containing R151A in the NBD (DnaK_R151A_) or K414I in the SBD (DnaK_K414I_) (*A*) or T199A and D481A or T199A and D481N in the SBD (*B*). As a reference for all experiments, the amplitudes of ATP-induced fluorescence changes in DnaK_wt_ (*A*) or DnaK_T199A_ (*B*) are shown. Data for DnaK_wt_ are the same as in Figure 3. Shown are the mean and standard deviation for at least three independent replicates. Statistical significance of differences to DnaK_wt_ was assessed using ordinary one-way ANOVA with Šídák’s multiple comparison; ∗, *p* < 0.05; ∗∗, *p* < 0.01; ∗∗∗, *p* < 0.001; ∗∗∗∗, *p* < 0.0001; no indications, differences are not significant. *C*, fluorescence traces for SBDβ-IB docking of DnaK_wt_, DnaK_R151A_, DnaK_D393A_, and DnaK_K414I_ (*left panel*) and DnaK_T199A_, DnaK_T199A,D481A_, DnaK_T199A,D481N_, and DnaK_T199A,D148A_ (*right panel*) at a 250s-timescale, showing that some of the mutants exhibit an increase in fluorescence after an initial rapid decrease. *D*, total amplitudes (*left panel*) and weighted rates (*right panel*) for the 0.25s-kinetics (open bars) and the 250s-kinetics (10–250 s only; filled bars) for all DnaK-variants that show an increase in fluorescence after the initial decrease (for details see Experimental Procedures). Shown are the mean and standard deviation for at least three independent replicates. The statistical analysis compares the absolute values for the amplitudes of the initial decrease (0.25s-kinetics) and the subsequent increase (10-250s-kinetics) separately for each DnaK-variant using ordinary one-way ANOVA with Šídák’s multiple comparison; ns, not significant; ∗∗, *p* < 0.01; ∗∗∗, *p* < 0.001; ∗∗∗∗, *p* < 0.0001.

For DnaK_R151A_ and DnaK_K414I_, we observed significantly lowered amplitudes for all ATP-induced conformational changes in the population average, consistent with the hypothesis that these residues are important for stabilizing the domain-docked state and their inability to release clients at elevated rates in response to ATP binding (Fig. 4*A*). In the population average, the kinetics for linker docking were accelerated, whereas the kinetics for SBDβ-IB docking and lid opening (steps 3 and 4 in Fig. 1*D*) were slowed down. Only the NBC closure occurred with kinetics similar to DnaK_wt_. Together, these observations are consistent with the idea that interdomain interface mutants do not perturb the mechanics of the NBD conformational dynamics and only affect the mechanics of SBDβ-IB docking and conformational changes that require successful docking of the SBDβ-IB, such as the opening of the lid. Moreover, DnaK_R151A_ overall displayed consistently lower amplitudes than DnaK_K414I_, suggesting that R151 is much more important than K414 for stabilizing the docking of the SBD on the NBD. Such an idea is supported by the observation that DnaK_R151A_ lid opening rates were reduced much more (ca. 1/2000^th^ relative to DnaK_wt_) than for DnaK_K414I_ (one-fourth relative to DnaK_wt_) (Fig. 4*A* right panel inset).

To investigate the conformational dynamics of the D481A and D481N replacements, we again had to introduce, in addition, the T199A replacement to avoid premature ATP hydrolysis. In the population average, DnaK_T199A,D481A_ displayed lowered amplitudes for SBDβ-IB docking and lid opening (Fig. 4*B*), in line with the inability of this mutant to release clients at elevated rates on ATP binding (Fig. S7) (12). In contrast to the above investigated interdomain interface mutants DnaK_R151A_ and DnaK_K414I_, DnaK_T199A,D481A_ exhibited no significantly reduced rate for SBDβ-IB docking in the population average. Interestingly, for DnaK_T199A,D481A_, we observed one-phase kinetics (Figs. S5 and S6), suggesting that the conformational equilibrium of the apo state with respect to SBDβ-IB docking was shifted towards a single conformation.

DnaK_T199A,D481N_, in contrast to DnaK_T199A,D481A_, displayed comparable amplitudes when compared to DnaK_T199A,_ consistent with its ability to release clients at elevated rates in response to ATP binding (Fig. S7). Strikingly, the kinetics for SBDβ-IB docking and lid opening for the DnaK_T199A,D481N_ variant were reduced to roughly 50% of the rates for DnaK_T199A_. Whether such differences cause differences in chaperone activity between eukaryotic Hsp70s and *E. coli* DnaK is currently not known.

Collectively, the results for DnaK_R151A_, DnaK_K414I_, DnaK_D481A_, and DnaK_D481N_ demonstrate that amino acid replacements in the interdomain interface alter the conformational equilibrium of Hsp70 between the docked and the undocked conformation and the mechanics of the docking process, explaining the high degree of conservation of these residues. Importantly, this shows that docking of the SBD onto the NBD is a prerequisite for opening of the lid and that this crucial conformational change is modulated throughout evolution to tune the conformational dynamics of Hsp70 chaperones, as has been suggested by Gierasch and colleagues (11).

### Amino acid replacements in the interdomain interface destabilize the ATP-bound state

**Figure 5:**
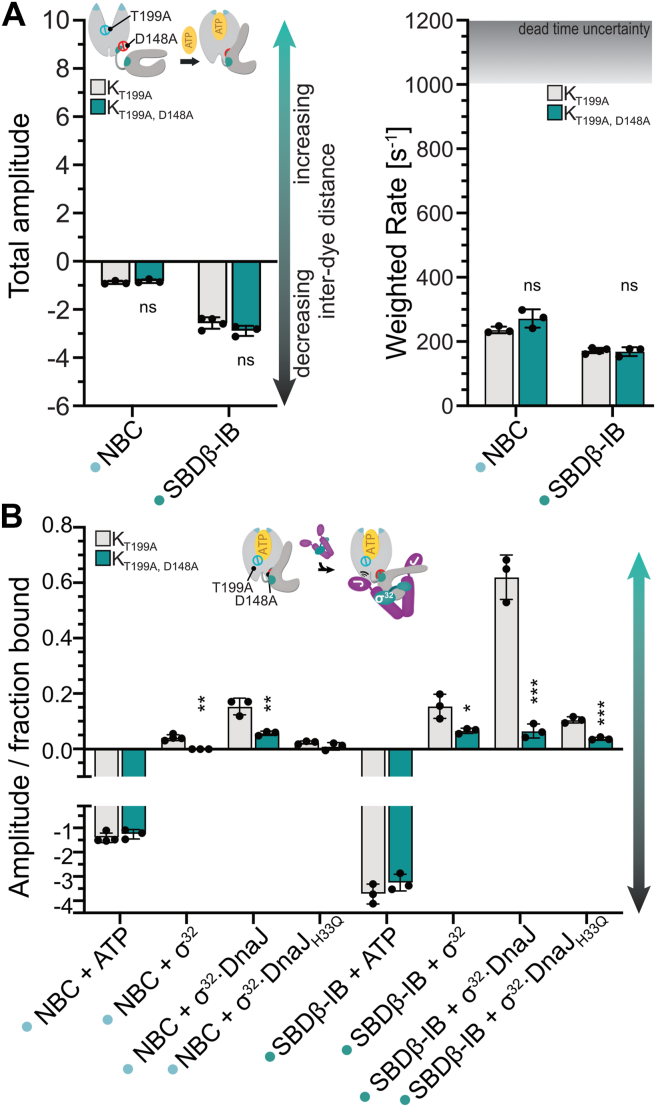
**The D148A amino acid replacement stalls client sent allosteric signals.***A*, total amplitudes (*left panel*) and weighted rates (*right panel*) for ATP binding to apoDnaK probes containing D148A and T199A in the NBD (Figs. S2 and S3, Movie S1, Movie S2). As reference, the amplitudes of ATP-induced fluorescence changes in DnaK_T199A_ are shown. Data for DnaK_T199A_ are the same as in Figures 3*B* and 4*B*. *B*, total amplitudes for conformational changes in apoDnaK_T199A_ and apoDnaK_T199A,D148A_ on binding of ATP and in DnaK_T199A_·ATP and DnaK_T199A,D148A_ATP on binding of the model client σ^32^ and the *E. coli* JDP DnaJ that binds and delivers σ^32^ to DnaK and synergistically with σ^32^ stimulates DnaK’s ATPase activity. Amplitudes were corrected for the fraction of DnaK in complex with the client using published K_d_ values. The DnaJ_H33Q_ variant is unable to stimulate DnaK’s ATPase activity but binds to σ^32^ and promotes its transfer to DnaK. Shown are the mean and standard deviation for at least three replicates. Statistical significance was assessed using ordinary one-way ANOVA with Šídák’s multiple comparison; ∗, *p* < 0.05; ∗∗, *p* < 0.01; ∗∗∗, *p* < 0.001; ∗∗∗∗, *p* < 0.0001; no indications, differences are not significant.

To monitor ATP-induced SBDβ-IB docking, we generally recorded the fluorescence changes for 0.25 s, which is sufficient to reach equilibration for DnaK_wt_ as previously established (17). When measuring the interdomain amino acid replacement variants (DnaK_D393A_, DnaK_R151A_, DnaK_K414I_, and DnaK_T199A,D481A_) for longer times (250 s) to capture any slower conformational changes, we observed that the initial rapid decrease was followed by an increase in fluorescence that was absent in DnaK_wt_, DnaK_T199A_, DnaK_T199A,D481N_, and DnaK_T199A,D148A_—proteins that were proficient in ATP-induced allosteric regulation (Figs. 4*C* and 5*A*). To elucidate the reason for this increase, we compared the absolute values of the amplitudes for the initial rapid decrease with the amplitudes of the subsequent slow increase (Fig. 4*D*). For DnaK_R151A_ and DnaK_D393A_, the amplitudes for the slow increase were larger than the initial fast decrease by 17- and 1.8-fold, respectively. In contrast, for DnaK_K414I_, the slow increase was slightly smaller (30%) than the initial rapid decrease. Only for DnaK_T199A,D481A,_ the amplitudes for initial decrease and subsequent increase were not significantly different. The difference in rates between the initial decrease in fluorescence and the subsequent increase was roughly 4 orders of magnitude and therefore well enough separated to be determined independently. As the SBDβ-IB variants report on the docking of the SBDβ onto the NBD, these results indicate that the distance between positions 80 in the NBD subdomain IB and 449 in the SBDβ increases again after the rapid docking of the SBDβ onto the NBD, which is incompatible with the ATP-bound domain-docked conformation.

**Figure 6:**
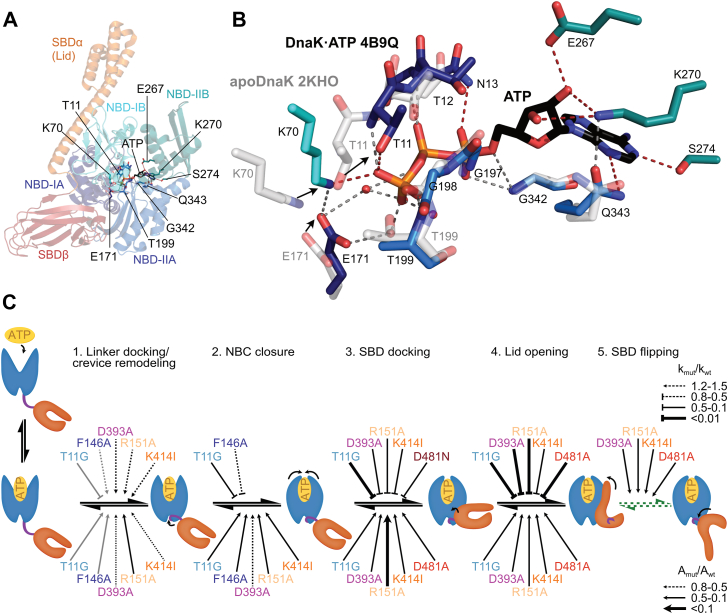
**A dynamic structural framework for the allosteric regulation of Hsp70 chaperones.***A*, sensing of ATP. Cartoon representation of DnaK·ATP (4B9Q) with ATP coordinating residues as sticks in atom colors with carbon in the color of the NBD subdomain: *dark blue*, IA; cyan, IB; marine, IIA; deep teal, IIB (Movie S1). *B*, zoom into the ATP binding pocked with ATP coordinating residues as sticks. Overlay (NBD-IIA aligned) of apoDnaK (2KHO; sticks in white, labels in *gray*) with DnaK·ATP (4B9Q; sticks colored as in A). *Graydashed lines* are polar contacts between backbone amides and ATP and between residue sidechains; *dark red* dashed lines are polar contacts between residue sidechain functional groups and ATP. Arrows indicate the movement on ATP binding. *C*, ATP-induced conformational changes start in the NBD with docking of the interdomain linker into the lower crevice which brings the SBD closer to the NBD (1). Concurrently, the crevice is remodeled, presumably to stabilize the docked linker. ATP binding brings the lobes of the NBD together resulting in NBC closure (2) and full exposure of the interdomain interface to allow SBDβ docking (3). Once the SBDβ is docked, the lid domain of the SBDα opens and docks onto the NBD (4). On substrate peptide binding, the SBDβ-NBD interface is destabilized and the SBD may flip by some 180° (5) as shown in the crystal structure of the ATP-and peptide bound state (7KRU; (15)). The arrows above the equilibrium arrows signify increased (pointed arrows) or decreased (blunt arrows) weighted rates of the amino acid replacement variants relative to DnaK_wt_ as indicated. The *arrows* below the equilibrium *arrows* indicate reduced total amplitudes relative to DnaK_wt_ as indicated. *Gray* arrows in Step (1) refer to crevice remodeling and *black arrows* to linker docking. The *green dashed* equilibrium *arrows* in step (5) indicate that this step does not occur in DnaK_wt_ in the absence of a substrate peptide (Fig. S3) but appears to occur even in the absence of a substrate peptide when amino acids that stabilize the SBDβ-docked state are replaced.

### The D148A replacement disrupts SBD-NBD signal transmission unidirectionally

Finally, we investigated how signals from Hsp70 clients are processed within Hsp70 to add to our understanding of the molecular mechanism that allows clients to stimulate the hydrolysis of ATP by Hsp70. We previously reported that DnaK_D148A_ exhibits wild-type-like ATP-induced substrate release rates, but its ATP hydrolysis rate is not stimulated by clients, suggesting a unidirectionally defective allosteric signaling mechanism where signals from the SBD cannot be processed in the NBD, but ATP-binding signals are normally transmitted to the SBD (12). DnaK_D148A_ is largely impaired in refolding chemically denatured firefly luciferase but does complement the temperature sensitivity phenotype of the *ΔdnaK E. coli* strain surprisingly well when produced at slightly elevated concentrations as compared to DnaK_wt_ (12). In the ATP-bound state, D148 is in the interdomain interface and forms a hydrogen bond with the peptide backbone of L484 in the SBDβ. L484 forms hydrophobic contacts with V440, which is located on β-strand 4 next to I438. The latter forms one side of the hydrophobic substrate binding pocket and directly interacts with clients. Together, these residues form a hydrophobic pathway that is proposed to transduce the allosteric signal from the client through the SBDβ toward the NBD (Figs. S2 and S3) (12, 13, 14, 15). To obtain insights into the structural mechanism underlying the disruption of the client signal transmission from the SBD to the NBD, we investigated the conformational dynamics of apoDnaK_D148A_ on ATP binding and of DnaK_D148A_·ATP on the binding of the model DnaK client σ^32^ in the presence or absence of its JDP cochaperone DnaJ. Briefly, together, DnaJ and DnaK bind the natively folded heat shock transcription factor σ^32^ in *E. coli* and relay it to the membrane-bound protease FtsH to initiate its degradation and thus establish the negative-feedback regulation of the heat shock response that controls the levels of chaperones, including DnaJ and DnaK (24, 27, 28, 29). Because σ^32^ and DnaJ together synergistically stimulate the hydrolysis of ATP by DnaK, we also included the T199A replacement to capture conformational changes in ATP-bound DnaK prior to ATP hydrolysis (30, 31). Moreover, we decided to limit the investigation of conformational dynamics of DnaK_D148A_ to the NBC and SBDβ-IB (Steps 2 and 3 Fig. 1*D*), because we recently showed that these changes are most instructive to understand the effects clients and JDPs have on the conformational dynamics of ATP-bound DnaK (17).

On ATP binding to DnaK_T199A,D148A_, NBC closure and SBDβ-IB docking occurred with amplitudes and rates that were not significantly smaller than the amplitudes and rates of DnaK_T199A_, demonstrating that ATP-induced conformational changes indeed are not perturbed by the D148A replacement (Fig. 5*A*). We showed recently that the binding of σ^32^ to DnaK_T199A_·ATP partially reverses ATP-induced conformational changes. DnaJ enhances these conformational changes when bound to σ^32,^ and for this effect, a functional J-domain is needed (17). Here, we reproduced these observations for NBC (opening) and SBDβ-IB (docking) (Steps 6 and 7, Fig. 1*D*). DnaJ_H33Q_, which is unable to stimulate DnaK’s ATPase activity but proficient in binding to σ^32^, lost its ability to enhance the reversion of ATP-induced conformational changes in DnaK_T199A_. On binding of σ^32^ to DnaK_T199A,D148A_·ATP, no fluorescence changes were observed for NBC in the absence of DnaJ or the presence of DnaJ_H33Q_. In the presence of DnaJ_wt_, the amplitudes of the DnaK_T199A,D148A_ fluorescence traces were significantly smaller than those of DnaK_T199A_ (Fig. 5*B*).

For σ^32^-induced conformational changes monitored with the SBDβ-IB variant, amplitudes of the fluorescence traces were significantly smaller in the absence and presence of DnaJ_wt_ or DnaJ_H33Q_. Thus, DnaK_T199A,D148A_ does not respond to client-binding with a conformational change in a similar extent to DnaK_wt_. We recently proposed that these conformational changes are important for interdomain communication without providing a formal proof (17). The DnaK variants used in this study substantiate this proposal with correlative evidence: Variants that are disturbed in signal transduction as detected by ATPase assays are also disturbed in the conformational changes.

Of note, the interdomain interface does not seem to be destabilized in this variant, as we did not detect any slow increase in fluorescence on long timescale measurements, contrasting findings for DnaK_R151A_, DnaK_D393A_, DnaK_K414I_, and DnaK_T199A,D481A_ (Fig. 4*C*).

Together, these data demonstrate that ATP-induced conformational changes occur unperturbed in DnaK_D148A_, whereas client-induced conformational changes are not fully executed and thus provide a structural framework for the unidirectionally defective allosteric signaling pathway in DnaK_D148A_. Beyond this, these data also show that the efficient reversion of ATP-induced conformational changes by a client and a JDP is a prerequisite to stimulate the hydrolysis of ATP by Hsp70 chaperones.

## Discussion

In this study, we obtained several insights into the allosteric mechanism of Hsp70 chaperones. (I) Of the ATP analogs tested, only ATPαS induced ATP-like conformational changes in DnaK, leading to a complete opening of the client-binding channel at a population-averaged rate of 64% of the wild-type rate. However, ATPαS is hydrolyzed by DnaK at about half the rate of ATP, and this hydrolysis is stimulated by a peptide substrate. All other tested ATP analogs only marginally opened the client-binding channel of DnaK, consistent with earlier publications (23, 32). (II) T11 is part of the ATP sensor, as previously proposed (25), that is necessary for rotation of the NBD subdomains in response to ATP binding and for relaying the presence of ATP to downstream processing by other key allosteric residues, such as F146, that stabilize the rotated subdomains of the ATP-bound NBD. (III) Stable docking of the interdomain linker into the lower crevice seems to be a prerequisite for stable docking of the SBD onto the NBD. (IV) Stable docking of the SBD onto the NBD is needed to stabilize the ATP-bound lobe-rotated state of the NBD and for efficient execution of conformational changes in the SBD. (V) Through D148, client binding induces a partial reversion of ATP-induced conformational changes in DnaK·ATP necessary for ATP hydrolysis.

ATP analogues are generally used to stabilize the ATP-bound state for extended periods of time for structural investigations or for demonstrating that a process depends on ATP binding but not hydrolysis. The nucleotides used in this study are clearly unsuitable for such investigations on DnaK, as they either do not convert DnaK completely into an ATP-like state or are hydrolyzed at significant rates. Interestingly, AMPPNP and ATPγS were able to elicit an allosteric signal all the way to the SBD, as evidenced by the rate of induced conformational change that was, for the ensemble average 31% (AMPPNP) and 52% (ATPγS) of the wild-type rate. As the amplitudes of the conformation changes are low, this indicates that the nucleotide analogues are unable to stabilize the ATP-like conformation. As detailed in Supporting Note 2, our data for AMPPNP and AMPPCP are consistent with structural observations.

How is ATP sensed? When comparing apo and ATP-bound states of full-length DnaK, it becomes clear that, on ATP binding, NBD subdomains IA and IB rotate relative to subdomains IIA and IIB. The adenine ring of the ATP is sandwiched between subdomains IIA and IIB coordinated by the sidechains of S274 (IIB) and Q343 (IIA) (Fig. 6, *A* and *B*; Movie S1). The ribose is coordinated by the sidechains of E267 and K270 (both in subdomain IIB). Backbone amide hydrogens of residues in subdomain IIA (G342, G197, G198, and T199) and in subdomain IA (N13, T12, T11) coordinate α, β, and γ-phosphates. Only two residues form polar contacts through their sidechains with the γ-phosphate, T11, and K70. Replacing these residues with residues that do not have functional groups capable of forming polar contacts, incapacitates the allosteric mechanism in Hsp70s (25, 26, 33). In contrast, replacing T199 in subdomain IIA with alanine does not compromise allostery in Hsp70s but greatly reduces ATPase rates (26), consistent with the observation that only backbone amide hydrogen of T199, and not the sidechain hydroxyl contacts the γ-phosphate of ATP. Thus, T11 (subdomain IA) and K70 (subdomain IB) are the key sensors of the γ-phosphate of ATP. Our data show that crevice remodeling (movements between subdomains IA and IIA) and linker insertion into the lower crevice occur at higher rates than NBC closure (movement between IB and IIB), suggesting that T11 makes the primary contact to the γ-phosphate as suggested previously (25) and K70 follows on the population average with a short delay. When comparing some 20 crystal structures of the isolated bovine Hsc70 NBD with many different amino acid replacements, including T13 variants (corresponds to T11 in DnaK) (PDB IDs 1BUP and 2BUP; (25)) and T204 variants (corresponding to T199 in DnaK) with bound ADP·P_i_ or ATP, no significant conformational differences could be observed (for details see Supporting Note 3), suggesting that the isolated NBD does not stably assume an ATP-like conformation or crystal contacts favor the apo/ADP-bound conformation. In all bovine Hsc70 NBD structures in complex with ATP where T13 or K71 (corresponding to K70 in DnaK) or both were present, the hydroxyl group of T13 formed hydrogen bonds to the γ-phosphate of ATP, whereas the ε-amino group of K71 was too far away to form a hydrogen bond. Here, we found that replacing T11 in DnaK with glycine reduced the rate of crevice remodeling and all subsequent conformational changes except linker insertion and reduced total amplitudes for all conformational changes (Fig. 3*A*). These data suggest that the rotation of subdomain IA relative to subdomain IIA is slowed down and that the rotated state of NBD lobe I relative to NBD lobe II is not stabilized. An NMR residual dipolar coupling study of the isolated NBD of bovine Hsc70 suggested shearing movements of all four subdomains of the NBD relative to each other (34). Thus, in the absence of nucleotides, the four subdomains of the NBD may oscillate relative to each other. Binding of ADP, as well as ATP, may stabilize the movement of subdomain IIB relative to IIA due to the coordination of the adenine and the ribose by residues in these subdomains. In addition, ATP stabilizes the rotated state of subdomain IA relative to IIA through contacts between T11 and the γ-phosphate, and, finally, subdomain IB relative to IA and IIB through contacts between K70 and the γ-phosphate. The position of K70 is further stabilized through polar contacts with E171 and hydrophobic contacts with F146 and P143 (Fig. S1, Movie S1). Consistently, replacing F146 with alanine had very little influence on the rates of conformational changes but destabilized the ATP-bound state with rotated subdomains IA and IB, as indicated by a greatly reduced total amplitude in our fluorescence traces (Fig. 3*B*). The ATP-induced NBD subdomain IA/IB-rotated conformation then allows docking of the SBD onto the NBD and, subsequently, lid opening. Therefore, the inability to stabilize this conformation is propagated in all subsequent conformational changes, reducing rates, and amplitudes of measured fluorescence changes (Fig. 3*A*).

How ATP induces linker insertion into the lower crevice is still enigmatic. Previously, we were unable to resolve the order of crevice remodeling and linker insertion (17). For DnaK_T11G_, linker insertion occurred with much higher rates than crevice remodeling, demonstrating that crevice remodeling is not a prerequisite for linker insertion. However, the total amplitude for the fluorescence changes of linker insertion were about half of the values for DnaK_wt_. Therefore, either the linker does not insert completely or the linker is not stabilized in the bound state, and dissociation rates of the linker are increased in DnaK_T11G_. There are two alternative not mutually exclusive explanations for these findings. In apoDnaK, linker association and dissociation could be in rapid equilibrium and ATP binding leads to trapping of the linker in the bound state, a process that is defective in the T11G variant. Alternatively, the signal that triggers linker insertion on ATP binding is transduced through another route. One route could be the backbone contacts of T11, T12 and N13, or T199, or both that cannot be accessed experimentally, as the backbone amide proton can only be replaced by altering respective amino acid with proline that is expected to cause severe structural defects. Another route could be the metal ions Mg^2+^ and K^+^ that were shown to be important for ATP hydrolysis. Mg^2+^ is coordinated by the β and γ-phosphate and four water molecules that are coordinated themselves by D8, E171, and D194. The bovine Hsc70 residues that correspond to E171 and D194 in DnaK (E175 and D199) have been implicated in allosteric regulation (35). However, defects in allosteric regulation of amino acid replacements in these residues could be rescued by an additional replacement in the α-helical lid of the SBD that facilitates lid opening. Thus, E171 and D194 contribute to force transmission between NBD and SBD. For DnaK_E171D_ and DnaK_E171Q_, we previously found 100-fold stimulation of peptide release by ATP and synergistic stimulation of the ATPase activity by DnaJ and σ^32^ (20), indicating that E171 is not absolutely essential for the allosteric mechanism. For D194 there is no obvious structural connection to the linker, but E171 sits on a restricted loop (N170-E171-P172-T173) and N170 and T173 interact with the linker residue D393 (Figs. 6*A* and S1). Thus, it is more likely that E171 contributes to allostery after linker docking by stabilizing the linker docked state. This hypothesis is supported by our finding that the D393A replacement has no reduced rates for linker docking but reduced total amplitudes of fluorescence changes. Previously, we showed that the isolated NBD of DnaK displays a 40-fold higher ATPase activity when the interdomain linker is present (amino acids 1–393) when compared to the NBD without the linker (amino acids 1–385), indicating that the linker insertion by itself favors the ATP hydrolysis-competent state (5). The D393A replacement in DnaK(1–393) displayed only a 12-fold elevated ATPase activity when compared to DnaK(1–385), suggesting that the polar contacts of D393 with N170 and T173 contribute to high ATPase activity but is not solely responsible for this. Replacing the hydrophobic residues of the linker (_389_VLLL_392_) with alanine residues completely abolishes the ATPase stimulation, underlining the importance of the insertion of V389 and L391 in between subdomain IA and IIA (5, 36).

The fact that neither linker docking rate nor NBC closure rates are decreased in DnaK_D393A_, whereas SBDβ-IB docking and lid opening rates are significantly decreased supports the proposed sequential order of ATP-induced conformational changes in DnaK (17). For amino acid replacements of the NBD-SBD interface-bridging residues R151 and K414 the linker-docking rates in the population average were also increased, and NBC closure rates were unaffected, whereas SBDβ-IB docking and lid opening rates were significantly decreased (Fig. 4*A*), supporting a sequential order of conformational changes. For the alanine replacement of the NBD-SBD interface-bridging residue D481, the rate of SBDβ-IB docking was only slightly reduced for the fast phase subpopulation and not at all for the population average, whereas lid opening rates were greatly reduced (ca. 1/640^th^ of wild-type rate). Thus, D481A does not affect the mechanism of SBDβ-IB docking. However, as DnaK_D481A_ cannot form a polar contact, found in DnaK_wt_ between the carboxylic group of D481 and the backbone amide of I168, it cannot lock in place the docked state, resulting in a low total amplitude of the recorded fluorescence changes (16% of wild-type amplitude; Fig. 4*B*). These data support a sequential order of SBDβ-IB docking and lid opening.

As mentioned above, only a subset of bacterial Hsp70s has aspartate in position 481. The majority of bacterial and most eukaryotic Hsp70s have asparagine in this position. Replacing D481 with asparagine reduced the SBDβ-IB docking rates to approximately one-half of the wild-type rate. The docked state, however, is locked in place presumably by polar contacts of the carbonyl of the asparagine sidechain amide group and the backbone amide hydrogen of I168, as evidenced by similar total amplitudes (Fig. 4*B*). The reduced SBDβ-IB docking rates are propagated to the lid as lid-opening rates are also reduced to approximately half of the wild-type rates. Whether such differences manifest in physiological consequences is currently unclear.

Surprisingly, for SBDβ-IB docking, all variants with destabilized NBD-SBD interface exhibited a slow increase in fluorescence after the initial rapid decrease in fluorescence. This was not observed for DnaK_wt_, or for variants with intact allostery like DnaK_T199A_ and DnaK_T199A,D481N_ (Fig. 5*E*). Of note, the DnaK_T199A,D148A_ variant, which is defective in the transmission of the substrate signal to the NBD but proficient in ATP-induced conformational changes (Fig. 5, *A* and *B*; (12)), also does not show said fluorescence increase (Fig. 4*C*).

ATP hydrolysis in DnaK_R151A_, DnaK_D393A_, and DnaK_K414I_ would lead to SBDβ-IB undocking and thus could account for the observed increase in fluorescence. However, as detailed in Supporting Note 4 ATP hydrolysis does not explain the observed changes. An alternative explanation for the slow increase in fluorescence could be the transition to a conformation different from the ATP and ADP-bound states. The most likely conformation is the recently published structures of DnaK in the presence of ATP and a substrate peptide (7KRU and 7KRW, called DnaK^S^ by Hendrickson and coworkers (15) in contrast to the DnaK·ATP conformation, 4B9Q, which was called DnaK^R^; compare Fig. S1 with Fig. S3). In the DnaK^S^ conformation the SBDα is released from the NBD and the entire SBD rotated by some 180° relative to the DnaK·ADP structure (2KHO) and still docked onto the NBD with an inserted linker like in the DnaK^R^ conformation (4B9Q). As the polar interactions between the sidechain of R151 and the backbone carbonyl of D481, between the sidechain carboxyl of D393 and the sidechain functional groups of N170 and T173, between the sidechain amino group of K414 and the sidechain carboxyl of D326, and between the sidechain carboxyl of D481 and the backbone amide of I168 that stabilize the NBD-SBD interface in the DnaK^R^ conformation are absent in DnaK^S^ (Fig. S3, pale cyan dashed lines in Movie S2), the respective amino acid replacements would destabilize DnaK^R^ but not impact DnaK^S^. In DnaK^S^, the distance between the residues that monitor SBDβ-IB docking is increased to 37 Å, as compared to 14 Å for DnaK^R^ (Table S1), which would relieve the quenching of our fluorophores (quenching limit ca. 15 Å). The distances for the crevice, linker and NBC variants are similar in DnaK^S^ as in DnaK^R^. So, we would not expect to notice the transition into the DnaK^S^ conformation when analyzing these variants. In DnaK^S^ the α-helical lid is closed over the SBDβ but not as much as in the crystal structure of the isolated SBD in complex with a substrate peptide (1DKX (37)) and the distance of our labeled residues (17 Å) is just outside the range for which fluorescence quenching is observed. Therefore, such a structure is unlikely to contribute significantly to the low amplitudes observed for lid opening in our allosteric variants. Since the rate of the initial decrease in fluorescence of SBDβ-IB docking is 3 to 4 orders of magnitude higher for the allosteric variants than the rate of the slow increase and the amplitude for the initial decrease is still only a fraction of the wild-type amplitudes, the majority of molecules in the domain-docked DnaK^R^ conformation will dissociate back to the conformation of apoDnaK and NBD and SBD may continue to reassociation and dissociate many times until occasionally a molecule converts into the DnaK^S^ conformation. Subsequently, ATP is hydrolyzed, ADP is dissociated, and the cycle restarts with ATP association. These findings are consistent with the hypothesis that substrate binding to DnaK^R^ leads to dissociation of the SBDβ from the NBD to allow back-rotation of the NBD lobes to a position that is optimal for ATP hydrolysis (12, 15, 16). The allosteric variants with amino acid replacements in the NBD-SBD interface simulate the substrate-induced destabilization of the NBD-SBD interaction and transit to the DnaK^S^ conformation without binding to a peptide, explaining their increased ATPase activity that is comparable to the ATPase rate of DnaK_wt_ in the presence of a substrate peptide. However, the rates for the DnaK^R^→DnaK^S^ transition do not correlate with the ATP hydrolysis rate for the different variants as mentioned in Supporting Note 4, indicating that the individual variants have additional defects in triggering γ-phosphate cleavage. The question is whether the transition to the DnaK^R^ conformation is tightly linked to client release. This does not seem to be the case for the allosterically defective variants analyzed here. For DnaK_R151A_ and DnaK_T199A,D481A_ the DnaK^R^→DnaK^S^ transition rates are about twofold higher than the lid opening rates, suggesting that these DnaK variants could transit through the cycle without releasing a bound peptide, which might result in futile cycles. For DnaK_D393A_ the DnaK^R^→DnaK^S^ transition rate is about half the lid opening rate (Table S3), suggesting peptide release before conversion into the DnaK^S^ conformation, which might contribute to the absent chaperone activity of this variant. Fig. 6*C* summarizes the influence of the different amino acid replacements on the conformational changes induced by ATP binding.

We recently published that peptide binding to DnaK·ATP reverses conformational changes induced by ATP binding to apoDnaK as measured by fluorescence changes for the different variants monitoring crevice remodeling, NBC closure, SBDβ-IB docking, and lid opening (17). Only the linker was not released by peptide binding. These data are in part consistent with the DnaK^R^→DnaK^S^ transition. In contrast, protein substrates only partially reversed the conformational changes, as evidenced by lower amplitudes of fluorescence changes. Here, we repeated these measurements for NBC closure and SBDβ-IB undocking and included the D148A amino acid replacement variant, shown previously to be unable to sense substrate binding. While DnaJ stimulates the ATPase activity of DnaK_D148A_, the protein substrate σ^32^ does not and no synergistic stimulation by DnaJ and σ^32^ is detectable (12). To prevent ATP-hydrolysis we included, as before, the T199A amino acid replacement. Whereas the fluorescence changes observed on ATP binding to apoDnaK_T199A,D148A_ were within experimental error identical to changes observed for DnaK_wt_, changes measured on binding of σ^32^ and DnaJ in complex with σ^32^ to DnaK_T199A,D148A_·ATP were significantly decreased. These data substantiate the hypothesis that the fluorescence changes observed are related to the conformation of the transition state for ATP hydrolysis (Fig. 5*B*). This is particularly striking for the fluorescence changes observed on binding of the DnaJ·σ^32^ complex. The magnitude of these fluorescence changes may depend on the client and possibly the JDP involved. It should be noted that in DnaK_wt_ the absolute value for the amplitude of fluorescence change observed on DnaJ·σ^32^ binding is significantly smaller than the absolute value of the amplitude observed on ATP binding (0.62 *versus* 3.72). These data indicate that the HiLyte labels remain well within the 15-Å-limit and, thus, are inconsistent with a full transition into the DnaK^S^ conformation, which would distance the labels by 37 Å. As σ^32^ stimulates the ATPase activity of DnaK much more efficiently than a peptide substrate (17), these data suggest that the peptide-induced conformational change of the SBD goes too far, beyond the optimal conformation for γ-phosphate cleavage and that the transition state is different from the DnaK^S^ conformation. How the conformation of the Hsp70 transition state looks awaits additional structural analysis.

## Experimental procedures

### Protein purification

Protein purifications were performed essentially as described previously (17) in detail.

#### DnaK

DnaK(2-606) variants (N-terminal methionine is cleaved *in vivo* by methionine aminopeptidase) were recombinantly produced as C-terminally His-tagged proteins in the *ΔdnaK E. coli* strain BB1994, incubated at 30 °C during all steps of protein production. Cells were harvested at 5000 g for 15 min at RT, and cell pellets were resuspended in 25 ml of chilled lysis buffer (20 mM Tris/HCl pH 7.9, 100 mM KCl, 1 mM PMSF, 5 mM MgCl_2_, 10 μg/ml DNase I, 8 μg/ml pepstatin, 10 μg/ml aprotinin and 5 μg/ml leupeptin) per 1.5 l of culture, snap frozen in liquid nitrogen, and stored at −20 °C. All subsequent steps were carried out at 4 °C or on ice if not indicated otherwise. After fresh protease inhibitors were added, cells were lysed with a MicroFluidizer (Avestin) and the obtained lysate was clarified by centrifugation at 20,000*g* for 30 min. The supernatant was mixed with 2 g of Protino Ni-IDA beads (Macherey-Nagel) and incubated for 20 min. The resin was subsequently collected in a gravity-flow column and washed first with 12.5 column volumes (CV) of wash buffer (20 mM Tris/HCl pH 7.9, and 100 mM KCl) followed by 6.25 CV of ATP buffer (wash buffer with 5 mM MgCl_2_ and 5 mM ATP/KOH, pH ∼7). To remove DnaK-bound clients, another 6.25 CV of ATP buffer was added, the flow was stopped, and the column was incubated for 30 min. Afterward, the flow was allowed to continue, and another 12.5 CV of wash buffer was added to remove residual ATP. The protein was then eluted in 2 ml fractions using elution buffer (20 mM Tris/HCl pH 7.9, 100 mM KCl, and 250 mM Imidazole/KOH pH 8). Protein-containing fractions were pooled and dialyzed against 2 l of dialysis buffer (40 mM HEPES/KOH pH 7.6, 150 mM KCl, 5 mM MgCl_2_) overnight. The next day the protein was aliquoted, snap-frozen in liquid nitrogen, and stored at −80 °C. If required, DnaK-bound nucleotides were removed by buffer exchanging into nucleotide removal buffer (50 mM Tris/HCl pH 7.5, 100 mM NaCl, 2 mM EDTA, 2 mM NaN_3_ and 10% glycerol) using Zeba spin columns (Thermo Fisher Scientific) and by treating the sample with calf intestine alkaline phosphatase (Roche, Basel; 2U per mg DnaK) at RT overnight. The next day, the buffer was exchanged into HKM buffer (25 mM HEPES/KOH pH 7.6, 150 mM KCl, and 5 mM MgCl_2_, using Zeba spin columns), samples were aliquoted, snap-frozen in liquid nitrogen, and stored at −80 °C.

#### DnaJ

DnaJ_wt_ used in this work was produced in the *E. coli* BL21 Rosetta strain and DnaJ mutants in *ΔdnaJ ΔcbpA E. coli* cells (WKG190) as N-terminal SUMO-fusion proteins and incubated at 30 °C during all steps of gene expression. Multiple transformed colonies were used to inoculate 100 ml of 2xYT medium supplemented with kanamycin (DnaJ_wt_) or ampicillin (DnaJ_H33Q_) (100 μg/ml) and used the next day to inoculate 3 l of 2xYT medium containing the appropriate antibiotic. The culture was grown until an OD_600nm_ of 1.2 was reached and induced with 0.2% arabinose (DnaJ_H33Q_) or 1 mM IPTG (DnaJ_wt_) and the protein was produced for 4 h. Cells were then harvested at 5000 g for 15 min at RT and cell pellets were resuspended in 25 ml of chilled lysis buffer (50 mM Tris/HCl pH 7.9, 500 mM NaCl, 0.6% Brij58, 5 mM MgCl_2_, 10 μg/ml DNase I, 1 mM PMSF, 8 μg/ml pepstatin, 10 μg/ml aprotinin, 5 μg/ml leupeptin) per 1.5 l of culture and snap frozen in liquid nitrogen and stored at −20 °C. All subsequent steps were carried out at 4 °C or on ice if not indicated otherwise. After addition of fresh protease inhibitors, cells were passed three times through a MicroFluidizer. The obtained lysate was clarified by centrifugation at 20,000*g* for 30 min. The supernatant was mixed with 1 g of Protino Ni-IDA beads and incubated for 20 min. The resin was subsequently collected in a gravity-flow column and washed first with 25 CV of wash buffer (50 mM Tris/HCl pH 7.9, 500 mM NaCl, 0.1% Brij58, 2 M Urea) and then with 25 CV of wash buffer with 1.5 M NaCl. To remove the detergent before dialysis, the resin was washed with 25 CV elution buffer (50 mM Tris/HCl pH 7.9, 500 mM NaCl, 2 mM 2-mercaptoethanol, 2 M Urea). The protein was then supplemented with another 4 ml of elution buffer, the flow stopped, and 4 mg of Ulp1 were added and the column sealed and incubated rotating for 2 h. After 2 h of incubation, Ulp1 was removed by addition of 0.16 g of Protino Ni-IDA beads and incubation for 20 min at 4 °C. The supernatant containing the protein was collected and the protein dialyzed against dialysis buffer (40 mM HEPES/KOH pH 7.6, 300 mM KCl and 10% glycerol) over night. The protein was then diluted to around 150 μM, aliquoted, snap frozen in liquid nitrogen, and stored at −80 °C.

#### σ^32^

σ^32^ was produced as SUMO-fusion protein in the *E. coli* BL21(DE3) derivative AR5088 lacking endogenous FtsH and containing the plasmid pACYClacIq (kindly provided by Dr Teru Ogura (38)) and incubated at 30 °C during all steps of gene expression. A multitude of freshly transformed AR5088 colonies were used to inoculate 150 ml of 2xYT medium supplemented with ampicillin (100 μg/ml) and used the next day to inoculate 4.5 l of 2xYT medium containing ampicillin. The culture was grown until an OD_600nm_ of 1.2 was reached, and protein production was induced with 1 mM IPTG for 3 h. Cells were harvested at 5000 g for 15 min at RT and cell pellets were resuspended in 65 ml chilled lysis buffer (20 mM Tris/HCl pH 7.9, 100 mM NaCl, 5 mM MgCl_2_, 0.05% NaDeoxycholate, 10 μg/ml DNase I, 1 mM PMSF, 8 μg/ml pepstatin, 10 μg/ml aprotinin, 5 μg/ml leupeptin) per 1 l of culture and snap frozen in liquid nitrogen and stored at −20 °C. All subsequent steps were carried out at 4 °C or on ice, if not indicated otherwise. Thawed cells were passed once through a MicroFluidizer after fresh protease inhibitors were added, and the obtained lysate was clarified by centrifugation at 20,000*g* for 30 min. The supernatant was mixed with 1 g of Protino Ni-IDA beads and incubated for 20 min. The resin was subsequently collected in a gravity-flow column and washed first with 25 CV of wash buffer (20 mM Tris/HCl, pH 7.9, and 100 mM NaCl) followed by 50 CV of ATP buffer (See DnaK purification) to remove σ^32^-bound DnaK. The beads were then washed with 25 CV of elution buffer (40 mM HEPES/KOH pH 7.6, 150 mM KCl, 2 mM 2-mercaptoethanol) to remove residual ATP. Subsequently, another 4 ml of elution buffer was added, the flow was stopped, and 1 ml of 4 mg/ml of Ulp1 protease dissolved in 40 mM HEPES/KOH pH 7.6, 150 mM KCl, 2 mM β-mercaptoethanol, and 50% Glycerol was added, and the closed column was rotated end-over-end for 2 h. To remove Ulp1, 0.16 g of fresh Protino Ni-IDA beads were added, and the column was rotated for 20 min. The eluate containing the protein was collected using gravity-flow, aliquoted, snap-frozen in liquid nitrogen, and stored at −80 °C.

### Preparation of HiLyte Fluor 488-labeled DnaK

Preparation of HiLyte Fluor 488 labeled DnaK was performed essentially as described previously (17). In detail: Proteins were thawed and aggregates removed by centrifugation at 20,000*g* for 15 min. The protein was then diluted to 2 mg/ml and a 500 μl aliquot supplemented with 10 mM ATP/KOH pH ∼7 and 50 mM freshly prepared DTT and incubated for 1 h at 30 °C. To remove DTT, buffer was exchanged twice against HKM (25 mM HEPES/KOH pH 7.6, 150 mM KCl, 5 mM MgCl_2_) using Zeba spin columns. For labeling, 5 μl of 42 mM HiLyte Fluor 488 dye (7.5- fold excess per cysteine, Anaspec, Fremont, USA) dissolved in water-free DMF were added and the reaction mixture incubated at RT for 1 h. To separate the protein from the unbound dye, the protein was separated on a self-packed Sephadex G50 (GE Healthcare) gelfiltration column (20 cm × 1 cm) equilibrated in nucleotide removal buffer (See DnaK purification). The red band corresponding to the labeled protein was collected and 2U of alkaline phosphatase were added to remove bound nucleotides by incubation at RT overnight. The next day, the buffer was exchanged against HKM using Zeba spin columns, labeling was confirmed by electrospray ionization mass spectrometry using a MaXis (Bruker), and the protein was snap-frozen in liquid nitrogen, and stored at −80 °C. Only 100% double-labeled protein was used for experiments.

### Preparation of fluorescently labeled peptides

Peptides were labeled with a fluorescent dye essentially as described previously (17). In detail: Lyophilized peptides were resuspended in water to a concentration of 5 mM or 1 mM depending on their solubility and stored at −20 °C. For labeling, 500 μM peptide (500 μl) was prepared in 100 mM HEPES, pH 7.6 and the peptides reduced by the addition of 1.5 M excess of TCEP and incubation for 30 min at 30 °C. Peptides were labeled with a 5-fold molar excess of 2-(4′-(iodoacetamido)anilino) naphthalene-6-sulfonic acid (IAANS, Molecular Probes, Invitrogen) for 2 h at RT. The peptide subsequently was separated from leftover dye on a self-packed G10 Sephadex column (20 × 1 cm) equilibrated in 100 mM HEPES, pH 7.6. Two drops per fraction were collected and only fractions containing the first fluorescent peak and displaying viscosity, while pipetting, were pooled, analyzed by high-resolution mass spectrometry, and stored at −20 °C.

### Kinetic fluorescence measurements

Kinetic fluorescence measurements were performed essentially as described previously (17). In detail: All kinetic measurements in this section were performed using an SX.18MV stopped-flow instrument at 30 °C (Applied Photophysics). Experiments with HiLyte Fluor 488 were performed at λ_ex_ = 488 nm and using a 530 nm cut-off filter. ATP-induced conformational changes were monitored after mixing 100 nM of HiLyte Fluor 488 labeled double-cysteine DnaK 1:1 with 10 mM ATP in HKM buffer. Client-induced conformational changes were monitored after mixing 100 nM HiLyte Fluor 488 labeled DnaK cysteine variants and 125 μM ATP 1:1 with 125 μM ATP in HKM buffer with either 10 μM σ^32^, or with 20 μM DnaJ variant, or with both 10 μM σ^32^ plus 20 μM DnaJ variant at 30 °C in the stopped-flow device. To compare the amplitude of the client-induced change with the amplitude induced by ATP binding, ATP-induced changes were recorded by mixing 100 nM of labeled DnaK 1:1 with 250 μM ATP in HKM buffer under similar conditions. Analysis of kinetic data was performed using Prism 9.5.1 (GraphPad). All traces for the 250s-timecourse of ATP binding to DnaK_SBDβ-IB_ (Fig. 5*E*) were y-transformed to start at 10 V by calculating the transformation constant from the Y_0_ value of the fit to the 0.25s-kinetics (Figs. 4*A*, 5, *A* and *C*, 6*A*) and the difference between the values for the 0.025s datapoint of the 0.25s- and 250s-kinetics. Data were generally fitted to exponential equations containing one to three exponential terms. Data of the 250s-kinetics (Fig. 5*E*) between 10 and 250 s were fitted to bi-exponential (DnaK_R151A_), bi-exponential with one stretched exponential term (DnaK_K414I_ and DnaK_D393A_), or tri-exponential (DnaK_D481A_) functions to yield satisfying fits (randomly distributed residuals).

### Peptide dissociation experiments

Peptide dissociation was recorded essentially as described previously (17). In detail: To determine peptide dissociation rates, 1 μM of nucleotide-free DnaK unlabeled double-cysteine variant or DnaK_wt_(2-606) was pre-incubated with 1 μM of pep65-C labeled with 2-(4′-(iodoacetamido)anilino) naphthalene-6-sulfonic acid (IAANS) in HKM buffer containing 5 mM DTT for 2 h at RT and subsequently mixing the complex 1:1 with quench buffer (50 μM pep65-C in HKM buffer containing 5 mM DTT) in a well of a 384 well plate (Corning, REF 3820) and recording the decrease in fluorescence emission in a CLARIOstar plate reader (BMG Labtech, Ortenberg, Germany; excitation: 335-15, dichroic: 376.2; emission: 420-20). ATP-induced peptide released was measured similarly except that fluorescence changes were recorded in the stopped-flow instrument (SX.18MV, Applied Photophysics; λ_ex_ = 335 nm, cut-off filter: 420 nm; 30 °C) after mixing 1:1 with quench buffer supplemented with 5 mM ATP.

### ATPase assay (“non-radioactive single turnover-type assay”)

To elucidate whether ATPαS is hydrolyzed by DnaK, the fluorescence signal changes within DnaK_linker_ (HiLyte Fluor 488-labeled DnaK_E217C,L392C_) on binding and hydrolysis of ATP or its analogues (ATPαS, AMPPNP) without and in presence of a peptide substrate (pep65) were recorded. The stocks of 4 μM DnaK_linker_, 4 μM ATP (Carl Roth, Karlsruhe, Germany) 4 μM ATPαS (Jena BioScience, Jena, Germany), 4 μM AMPPNP (Jena BioScience, Jena, Germany) and 4 μM ATP-free ADP (as a negative control) were prepared using HKM-buffer (40 mM HEPES/KOH pH 7.6, 150 mM KCl, 5 mM MgCl_2_) with 0.05% Triton X-100. The components of the reaction were pipetted in the following order with the final concentration in 384-well microtiter plates (Corning, Kennebunk, USA; REF 3820): buffer, 1 μM DnaK, 1 μM ATP (or analogues) and 10 μM pep65. The change in fluorescence was monitored in the CLARIOstar plate reader (BMG Labtech, Ortenberg, Germany) at 22 °C with the following optics settings: excitation filter 488-14; emission filter 535-30; gain 800, gain adjustment using the well DnaK + ADP with the target value of 70%.

### Statistical analysis

Of note, except for Fig. 2, in which only apoDnaK_wt_ was mixed with different ATP analogs, in all other figures, each bar represents a different protein, independently purified, labeled with HiLyte Fluor 488, and independently measured. There is, therefore, no intrinsic link between the individual bars within a figure. For the data of the crevice variant, only two pairs of measurements are compared (DnaK_T11G_ with DnaK_wt_ and DnaK_T199A,F146A_ with DnaK_T199A_); therefore, simple Student’s *t* test was sufficient to establish significance of differences. In contrast, for all other conformational changes (Linker, NBC, SBDβ-IB, and Lid) in Figure 3, Figure 4, Figure 5 the datapoints for each of the wild-type DnaK variants that are shown for comparison are the same data. For these variants, ordinary one-way ANOVA with Šídák’s or Dunnett’s multiple comparison was used to establish significance of differences. Thereby, the data for each of the allosteric variants were compared to the data for DnaK_wt_ irrespective of whether they are shown in one figure or in different figures (*e.g.* DnaK_T11G,Linker_, DnaK_D393A,Linker_, DnaK_R151A,Linker_, and DnaK_K414I,Linker_ are compared with DnaK_wt, Linker_ irrespective of the fact that they are shown separately in Figs. 3 and 4). It should also be noted that the variance of the experiment depended on the individual conformational changes measured and is different for every pair or triplet of bars shown within one figure, as amplitudes and rates were more difficult to determine accurately when the amplitudes were very small or the rates were close to the deadtime uncertainty of the instrument.

## Data availability

All data are contained within the manuscript and supporting information. Raw data are available from the lead contact upon request.

## Contact for reagent and resource sharing

Further information and requests for resources and reagents should be directed to and will be fulfilled by the Lead Contact, Matthias P. Mayer (m.mayer@zmbh.uni-heidelberg.de).

## Supporting information

This article contains supporting information (5, 7, 8, 12, 15, 17, 18, 19, 20, 25, 26, 32, 39).

## Conflict of interest

The authors declare that they have no conflicts of interest with the contents of this article.
