# Supplementary material for: A dynamic structural framework for the allosteric regulation of Hsp70 chaperones
Source: J Biol Chem. 2025 Jul 24;301(9):110516. doi: 10.1016/j.jbc.2025.110516 (PMC12406271; doi:10.1016/j.jbc.2025.110516)
Supplement: Supporting Information [file mmc3.pdf]

A dynamic structural framework for the allosteric regulation of Hsp70 chaperones

Lukas Rohland, Roman Kityk, Luka Smalinskaitė, Veronika Lashkul, and Matthias P. Mayer*

* to whom correspondence should be addressed at: m.mayer@zmbh.uni-heidelberg.de

Supporting information

Supporting Notes

Supporting Tables S1-S3

Supporting Figures S1 – S7

Legends for Movie S1 and S2

References for SI

Supporting notes

Supporting Note 1: Discussion of the fast phase subpopulation as compared to the population average

As mentioned in the main text, we previously found that the individual conformational changes (linker docking, crevice remodeling, NBC closure, SBDβ-IB docking and lid opening) elicited by ATP binding to apoDnaK occur with triphasic kinetics. As discussed in detail previously ((1) supplemental information), the most likely reason for these kinetics is the coexistence and presumably continuous interconversion of different conformers in apoDnaK that bind ATP with different association rates constants. Whether these distinct DnaK conformers differ in their ability to refold denatured proteins or whether they even cooperate in the process is currently unknown and awaits the discovery of amino acid replacement variants that separates the individual conformers. To elucidate whether there is any foundation for such speculations and whether individual conformers behave like the population average or distinct from it, we also analyzed the fast phase kinetics.

Binding of ATP analogues

Analysis of the fast phase of the nucleotide association overall paints a similar picture as the population average, with the exception that in the fast phase subpopulation the amplitude for ATPαS-induced lid opening was significantly decreased as compared to ATP-induced opening (1.36 ± 0.08 vs. 1.74 ± 0.07; $p = 0.0076$) but was not significantly different in the population average (5.02 ± 0.14 vs. 5.89 ± 0.62; $p = 0.105$). In contrast, the differences in rates were not statistically significant for the fast phase subpopulation (22.8 ± 4.1 s^{-1} vs. 32.7 ± 3.6 s^{-1}; $p = 0.16$) but significant in the population average (7.05 ± 0.82 s^{-1} vs. 11.09 ± 0.44 s^{-1}; $p = 0.0039$). (**Figs. 2B** and **S4A**).

$DnaK_{T11G}$

For the fast phase subpopulation of $DnaK_{T11G}$, we made similar observations as for the weighted average of the entire population except for the variant that monitors the crevice. This variant displayed a similar amplitude as $DnaK_{wt}$ for ATP-induced crevice remodeling but a lower crevice remodeling rate (**Fig. S4B**).

$DnaK_{T199A,F146A}$

For the fast phase conformer of $DnaK_{T199A,F146A}$, crevice remodeling occurred with decreased amplitude but at a wild-type-like rate, whereas NBC closure exhibited a wild-type-like amplitude but a reduced rate (**Fig. 3D**), suggesting that the respective subpopulation adopts a conformation where F146 may be important for the mechanics of NBC closure.

$DnaK_{D393A}$

A surprising result for $DnaK_{D393A}$ was the increased linker docking rate at the population average as compared to $DnaK_{wt}$. The accelerated linker docking in the population average is a result of a shift in the conformational equilibrium of $apoDnaK_{D393A}$ with a reduction of the subpopulations that contribute the medium and slow phase to the kinetics as compared to $DnaK_{wt}$, allowing the fast phase conformers in the $DnaK_{D393A}$ populations to contribute much more to the population average than in $DnaK_{wt}$ (**Fig. S6**). These data suggest that replacements

of residues involved in allosteric signaling also affect the ground state equilibria before ATP binding. When we compared the fast phase rates of DnaK$_{D393A}$ and DnaK$_{wt}$ with each other (**Fig. S4D**; 776 ± 13 s^{-1} vs. 789 ± 13 s^{-1}), it became apparent that in DnaK$_{D393A}$ linker docking occurred at rates that were not significantly different from the rates of DnaK$_{wt}$. Besides this, the fast phase conformer only differed from the population average in that it no longer displayed a significant amplitude difference for NBC closure on ATP binding, in line with the idea that the NBD dynamics are still intact in DnaK$_{D393A}$.

DnaK$_{R151A}$ and DnaK$_{K414I}$

As for DnaK$_{D393A}$, the accelerated linker docking kinetics for DnaK$_{R151A}$ and DnaK$_{K414I}$ in the population average are a result of a reduced fraction of conformers responsible for medium and slow phases for these mutants which causes the fast phase conformer to dominate the population average. The fast phase subpopulation displays similar reductions in amplitudes when compared to the total population average, with the exception of linker docking and NBC closure which seem to occur mostly unperturbed in DnaK$_{K414I}$ (**Fig. S4E**). The kinetics for the fast phase subpopulation were also comparable to the total population average, with the exception of linker docking, which occurred with slightly lower rates in DnaK$_{R151A}$ than in DnaK$_{wt}$.

DnaK$_{T199A,D481A}$ and DnaK$_{T199A,D481N}$

For DnaK$_{T199A,D481A}$ the fast phase subpopulation displayed a similar reduction in amplitudes for SBDβ-IB docking and lid opening as the population average (**Fig. S4F**). The SBDβ-IB docking and lid opening-kinetics of the fast phase subpopulation of DnaK$_{T199A,D481A}$, however, were significantly slowed down as compared to DnaK$_{wt}$, suggesting that D481 is important for the mechanics of SBDβ-IB docking. This is missed in the analysis of the weighted average of the population as the kinetics of DnaK$_{T199A,D481A}$ is monophasic missing medium and slow phase (**Fig. S6**) that reduce the average rate in DnaK$_{wt}$.
DnaK$_{T199A,D481N}$ displayed comparable amplitudes for both the population average and fast phase conformer when compared to DnaK$_{T199A}$. Strikingly, the kinetics for SBDβ-IB docking (total population 88.0±1.3 s^{-1} vs. 172.1±4.2 s^{-1}, fast phase 148.5±6.8 s^{-1} vs. 240.9±14.6 s^{-1}) and lid opening (population 6.5±0.1 s^{-1} vs. 14.4±0.6 s^{-1}, fast phase 28.7±0.9 s^{-1} vs. 56.3±2.1 s^{-1}) in the population average and fast phase conformer for the DnaK$_{T199A,D481N}$ variant were reduced to roughly 50% of the rates for DnaK$_{T199A}$.

DnaK$_{T199A,D148A}$

On ATP-binding to apoDnaK$_{T199A,D148A}$ NBC closure and SBDβ-IB docking occurred with similar rates and amplitudes as for apoDnaK$_{wt}$ for the population average and also for the fast, medium and slow phase subpopulations (**Figs. S4G, S5 and S6**), indicating that even down to the subpopulations, DnaK$_{T199A,D148A}$ responds to ATP like DnaK$_{wt}$.

Supporting Note 2

There are no structures of DnaK with bound ATP analogues. However, there are structures of the NBD of bovine Hsc70 in complex with AMPPNP, of *Cryptosporidium parvum* cytosolic Hsc70 in complex with AMPPNP, and of human BiP in complex with AMPPNP and AMPPCP that could give hints why these ATP analogues did not convert DnaK into the ATP-bound

conformation. In two of the AMPPNP-structures (CpHsc70-NBD, PDB ID 3KVG; hBiP, 3LDO) the γ-phosphate of AMPPNP is in a very similar position as the γ-phosphate of ATP in the divers Hsp70 structures (DnaK: 4B9Q, 4JN4; Hsc70-NBD: 1KAX, 2BUP; and BiP: 5E84), making contacts with the equivalent of T11, consistent with the eliciting of the allosteric signal. In contrast, in the bovine Hsc70-NBD structure (2QWR) the γ-phosphate of AMPPNP was displaced and did not form polar contacts to T13 or K71, apparently unable to stabilize the ATP-like conformation. As the ATP binding pocket and all ATP-interacting residues are conserved (except N13 in bacterial Hsp70s that is Y in eukaryotic Hsp70s), these structures might suggest that AMPPNP can assume different conformations in the ATP-binding pocket at least of some Hsp70s and the time spent in the conformation with the γ-phosphate in the ATP-like position may depend on less conserved parts of the protein. Interestingly, none of the NBD structures in complex with AMPPNP contained Mg^{2+} in the active site, although $Mg^{2+}$was present during crystallization (in two of the three structures). The conformation of AMPPNP seems to be stabilized by a network of water molecules involving the hydrogen of the imidogroup of AMPPNP. Such a configuration seems more flexible than the configuration with coordinated Mg^{2+}. In contrast, in the structure of BiP-NBD in complex with AMPPCP (5F2R) Mg^{2+} was well coordinated but the γ-phosphate was displaced as compared to the γ-phosphate in the ATP-bound structures. Nevertheless, the γ-phosphate of AMPPCP formed hydrogen bonds with T37 (equivalent to T11) and K96 (eq. K70) thereby stabilizing the ADP-like conformation. Such a conformation would be consistent with our observations that the rate of the fluorescence change on binding of AMPPCP were very low. However, some flexibility of the NBD should be possible as a slow partial opening of the lid is observed.

Supporting Note 3

To analyze the difference between structures of isolated Hsp70 NBDs in the ADP bound state and structures in the ATP bound state, all available bovine Hsc70 NBDs in complex with ADP or ATP were aligned to the first solved crystal structure (PDB ID 1ATR). The 14 crystal structures of the NBD of bovine Hsc70 with many different amino acid replacements, including T13 variants (corresponds to T11 in DnaK) (PDB IDs 1BUP and 2BUP; (2)) and T204 variants (corresponding to DnaK-T199, 1ATS (3)), that contained ADP or ADP·P_i aligned to 1ATR with an average root mean square deviation (RMSD) of 0.829 ± 0.141 Å (all atoms no outlier rejection). Six crystal structures of bovine Hsc70 that contained ATP aligned to 1ATR with an average RMSD of 0.829 ± 0.164 Å. Thus, NBD structures that contained ATP aligned with the ADP containing NBD structure 1ATR with the same RMSD as ADP containing structures. Similarly, alignment of the NBDs of DnaK in the ATP conformation results in an RMSD of 1.082 Å. In contrast, alignment of the NBD of DnaK·ATP with DnaK·ADP results in an RMSD of 3.976 Å. These data suggest that the isolated NBD cannot stably assume the NBD lobe rotated conformation or that crystallization of the isolated NBD preferentially selects the apo/ADP bound conformation.

Supporting Note 4

When measuring SBDβ-IB docking for the domain interface amino acid replacement variants $DnaK_{R151A}$, $DnaK_{D393A}$, and $DnaK_{K414I}$ we observed a slow increase in fluorescence that followed the initial rapid decrease. As $DnaK_{R151A}$, $DnaK_{D393A}$, and $DnaK_{K414I}$ have an increased

ATPase rate (5-, 12-, and 26-fold relative to $DnaK_{wt}$, respectively); (4-6), one could argue that ATP is hydrolyzed in these variants and DnaK converts from the ATP-bound to the ADP-bound conformation with concomitant dissociation of the SBDβ from the NBD. If this were the case, one would expect the following: (I) The rate of the fluorescence increase should be similar to the published ATP hydrolysis rates of the respective DnaK variant. This is at least for $DnaK_{R151A}$ (ATPase: 0.0028 s^{-1} (4), fluorescence increase: 0.014 s^{-1}) and $DnaK_{D393A}$ (0.0074 s^{-1} (5) versus 0.023 s^{-1}) not the case. Only for $DnaK_{K414I}$ the rates are similar (0.016 s^{-1} (6) versus 0.013 s^{-1}). (II) ADP dissociation rates under the conditions used (0.022 s^{-1}; (7)) are higher than ATP hydrolysis rates of the variants and any ADP produced by ATP hydrolysis is replaced by ATP which was present at a final concentration of 5 mM. At steady-state, only about 11% ($DnaK_{R151A}$), 25% ($DnaK_{D393A}$), and 41% ($DnaK_{K414I}$) of the DnaK molecules could be in the ADP-bound state, and the ratio of amplitudes of the fluorescence increase divided by the amplitude of the initial decrease should be in a similar range as the relative fraction of ADP-bound conformation. This is not the case for $DnaK_{R151A}$ (ratio of the slow increase divided by the initial decrease = 17 versus 0.11 for fraction of ADP-bound molecules) and $DnaK_{D393A}$ (1.8 versus 0.25). Only for $DnaK_{K414I}$ the ratio is within a reasonable range of the expectation (0.7 versus 0.41) (**Fig. 5F**). (III) The lid would close again on ATP hydrolysis, and the actually observed difference between final and starting fluorescence should reflect that fraction of molecules in the ATP-bound state. This is definitely not the case for any of three variants ($DnaK_{R151A}$: total amplitude of mutant divided by total amplitude of wild type = 0.13, fraction in ATP state 0.89; $DnaK_{D393A}$: 0.12 vs. 0.75; $DnaK_{K414I}$: 0.11 vs. 0.55). Moreover, $DnaK_{T199A,D481A}$, which cannot hydrolyze ATP, also exhibited the slow fluorescence increase. Thus, ATP hydrolysis is not the cause for the slow increase in fluorescence. The more likely hypothesis seems to be isomerization into a conformation that is similar to the DnaK·ATP·peptide structure (PDB ID 7KRU, (8) as discussed in more details in the main text.

SI Tables and Figures

Table S1: ATP and peptide-induced conformational changes in Hsp70s. Distances between Cβ atoms of DnaK double cysteine variants in the ADP bound, SBD closed, domain-undocked conformation (PDB ID 2KHO, (9)), the ATP bound, SBD open, domain-docked conformation (PDB ID 4B9Q, (10)), and the ATP & peptide bound, SBD closed, domain docked conformation (PDB ID 7KRU, (8)) were determined with the Pymol software (Vers. 1.8.6). This table is reproduced from (1)

**Double cysteine variant**		**Cβ distance in Å**		
		**2KHO**[a]	**4B9Q**	**7KRU**
Crevice	T22C-E213C	19	16	17
Linker	E217C-L392C	27	4	4
NBC	S87C-R253C	21	10	12
SBDβ-IB	E80C-A449C	67	14	37
Lid	E430C-R547C	12	88	(17)[b]

a, the 2KHO structure is a composite of the 1DKG crystal structure of DnaK's NBD in complex with GrpE and the 1DKX crystal structure of DnaK's SBD in complex with the NR-peptide, aligned according to residual dipolar coupling and spin-labeling NMR experiments. Therefore, interdomain distances (Linker and SBDβ-IB) and the NBC distance are less accurate.

b, distance measurement from the 7KRW structure as residues beyond 536 are not resolved in the 7KRU structure. Since the resolution of the 7KRW structure is only 7.7 Å the uncertainty of this distance measurement is very high.

Table S2: Biochemical properties of the amino acid replacement variants used in this study
ATPase activity of wild type DnaK and stimulation factors for DnaJ, substrate (σ^{32}) and DnaJ+substrate stimulated ATPase activity, stimulation factor for ATP-stimulated peptide release, and ATP-induced conformational changes as measured by blueshift (in nm) of the emission maximum of tryptophane fluorescence. All ATPase and peptide release values for amino acid replacement DnaK proteins are $\log_2$ transformed as detailed in footnotes. Heat map highlights the deviations (negative, red; positive, blue) relative to $DnaK_{wt}$ in two-fold steps. Conformational changes for DnaK variants are blueshift of tryptophane fluorescence relative to $DnaK_{wt}$ or qualitative information as available from the references.

replace-ment	ATPase basal[c]	ATPase (K+J)[d]	ATPase (K+S)[e]	ATPase (K+J+S)[f]	$k_{off,pep}$ +ATP[g]	Conformation[h]	References
$DnaK_{wt}$	$6{\cdot}10^{-4}s^{-1}$	2.37	2.09	47.02	1177	-3.45 nm	(4-6)
T37G[a]	-2.38*		-		-	- (prot)	(11)
T13G[b]	-8.84					no ΔRg	(2)
T11G					-6.91		This study
F146A	2.24	-0.12	-0.68	-1.48	-5.22	0.43	(6)
D148A	1.77	0.23	-0.92	-3.62	1.29	1.16	(6)
R151A	1.45	-1.30	-1.77	-5.15	-8.98	-0.01	(4)
T199A	-5.04			-4.30	-0.48	wt blueshift	(12) this study
D393A	3.62	-0.81	-1.25	-5.23	-7.12	0.07	(5)
K414I	4.69		-			no blueshift	(6) (13)
D481A	6.39	-1.57	-1.45	-5.51	-8.82	-0.03	(6)
D481N					-1.00		This study

a: *Cricetulus griseus* BiP (T37 corresponds to T11 in *E. coli* DnaK)
b: *Bos taurus* Hsc70 (T13 corresponds to T11 in *E. coli* DnaK)
c: basal ATPase rate: $DnaK_{wt}$, single turnover ATPase rate in s^{-1}; variants, $\log_2$-transformed ATPase rates relative to $DnaK_{wt}$: $y = \log_2\left(\frac{k_{hyd,mut}}{k_{hyd,wt}}\right)$ (single turnover ATPase; except *, steady state ATPase)
d: ATPase rate stimulated by 50 nM DnaJ: $DnaK_{wt}$, stimulation factor $y = \frac{k_{hyd,wt+J}}{k_{hyd,wt}}$; variants, $\log_2$-transformed stimulation factors relative to $DnaK_{wt}$: $y = \log_2\left(\frac{k_{hyd,mut+J}/k_{hyd,mut}}{k_{hyd,wt+J}/k_{hyd,wt}}\right)$ (single turnover ATPase)
e: ATPase rate stimulated by 1 µM σ^{32}; $DnaK_{wt}$, stimulation factor $y = \frac{k_{hyd,wt+S}}{k_{hyd,wt}}$; variants, $\log_2$-transformed stimulation factors relative to $DnaK_{wt}$: $y = \log_2\left(\frac{k_{hyd,mut+S}/k_{hyd,mut}}{k_{hyd,wt+S}/k_{hyd,wt}}\right)$ (single turnover ATPase); -, not stimulated by a peptide substrate (11);
f: synergistic stimulation by 50 nM DnaJ + 1 µM σ^{32}: $DnaK_{wt}$, stimulation factor $y = \frac{k_{hyd,wt+J+S}}{k_{hyd,wt}}$; variants, $\log_2$-transformed stimulation factors relative to $DnaK_{wt}$: $y = \log_2\left(\frac{k_{hyd,mut+J+S}/k_{hyd,mut}}{k_{hyd,wt+J+S}/k_{hyd,wt}}\right)$ (single turnover ATPase)
g: ATP-stimulated substrate release; $DnaK_{wt}$, ATP-stimulated dissociation rate relative to unstimulated dissociation rate $y = \frac{k_{off,wt+ATP}}{k_{off,wt}}$; variants, $\log_2$-transformed relative stimulation factors: $y = \log_2\left(\frac{k_{off,mut+ATP}/k_{off,mut}}{k_{off,wt+ATP}/k_{off,wt}}\right)$; -, no ATP stimulation detectable;
h: ATP-induced conformational changes as determined by proteolysis (prot), small angle X-ray scattering (SAXS) (ΔRg, change in radius of gyration); or tryptophane fluorescence (blueshift; $\Delta\lambda = \lambda_{max,+ATP} - \lambda_{max,-ATP}$); $DnaK_{wt}$, $\Delta\lambda$ is shown; variants, $y = \frac{\Delta\lambda_{mut}}{\Delta\lambda_{wt}}$; -, no ATP induced conformational change.

Table S3: Weighted rates of ATP-induced fluorescence changes in $DnaK_{wt}$ and DnaK amino acid replacement variants.

Amino acid replacement	Crevice	Linker	NBC	SBDβ-IB (0.25 s)	SBDβ-IB (250 s)	Lid
wt	164.2 ± 44.2	442.5 ± 19.4	297.1 ± 47.4	156.5 ± 4.7	nd	13.8 ± 0.8
T11G	22.4 ± 4.8	395.9 ± 23.3	109.8 ± 22.5	35.3 ± 0.6	nd	0.021 ± 0.001
R151A		653.4 ± 17.4	356.7 ± 129.1	79.3 ± 8.2	0.014 ± 0.006	0.007 ± 0.002
D393A		610.4 ± 46.0	376.0 ± 72.1	67.8 ± 7.4	0.023 ± 0.006	0.043 ± 0.003
K414I		624.0 ± 5.0	376.5 ± 80.1	31.5 ± 2.6	0.0132 ± 0.0005	3.61 ± 0.51
T199A	260.3 ± 32.7		236.7 ± 10.2	172.1 ± 8.3	nd	14.35 ± 1.15
T199A,F146A	323.1 ± 60.5		248.4 ± 57.2			
T199A,D148A			272.0 ± 28.5	169.0 ± 13.9	nd	
T199A,D481A				159.0 ± 6.5	0.058 ± 0.015	0.022 ± 0.001
T199A,D481N				88.0 ± 7.3	nd	6.51 ± 0.16

Shown are mean and standard deviation; nd, fluorescence increase not detected;

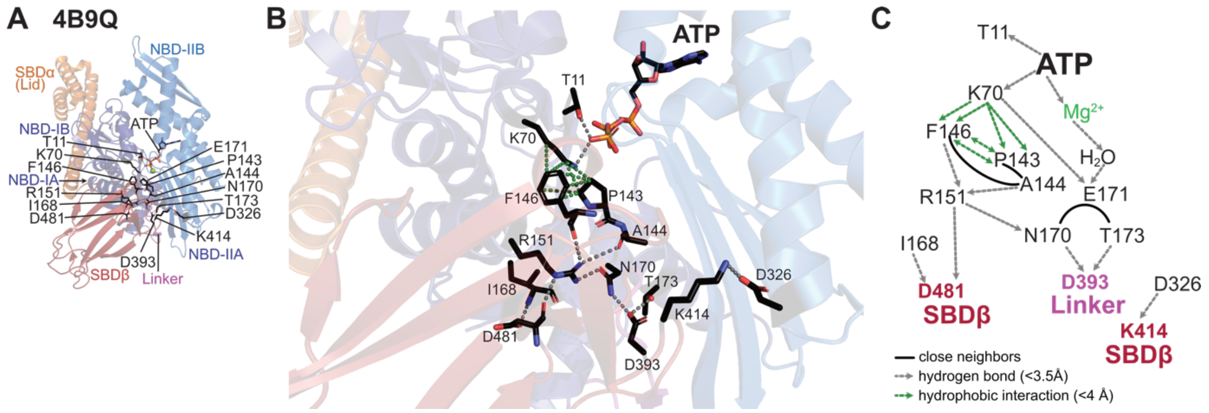

Figure S1. Allosteric signaling pathway for ATP-induced conformational changes.

A) Overview over the ATP allosteric signaling pathway. Shown is the ATP-bound structure of DnaK (PDB ID 4B9Q) in cartoon representation and residues that have been demonstrated to be important to process ATP-related allosteric signals in Hsp70 chaperones and are crucial to allow Hsp70 to adopt the ATP-bound state as sticks. Taken from Figure 1. Nucleotide binding domain (NBD) lobe I in dark blue; NBD lobe II in marine blue; substrate binding domain β-sandwich subdomain (SBDβ) in dark red; α-helical lid domain (SBDα) in orange; residues in atom colors with carbon in black.

B) Zoom into the ATP allosteric signaling pathway. The pathway starts in the NBD with recognition of the γ-phosphate of the ATP by T11 and K70. The signal is then picked up by P143 and relayed to R151 on the surface of the NBD forming the interface with the SBDβ. F146 is thought to stabilize P143 in its ATP state conformation. Conformational changes in the NBD cause retraction of the interdomain linker and remodeling of the lower crevice, presumably to promote linker retention. The docked linker is stabilized by N170 and T173 which both contact the linker residue D393 (E171, which interacts with K70 and water molecules in the coordination sphere of Mg^{2+} is left away for clarity). As a consequence of linker docking, two things happen. 1) The nucleotide binding cleft closes and 2) docking of the linker shortens the interdomain distance and brings the SBD in close proximity of the NBD. Together, both rearrangements allow docking of the SBD onto the NBD. The docked SBD is stabilized on the NBD by a clamp formed by polar interactions of D481 with the backbone amide of I168 on NBD lobe I and by polar interaction of K414 with D326 on NBD lobe II.

C) Simplified overview over the ATP allosteric signaling pathway. Green dashed lines indicate hydrophobic interactions (C-C-distance, <4.2Å), gray dashed lines electrostatic interactions (O-O or N-O-distance <3.5Å), and black solid lines connection through the peptide backbone.

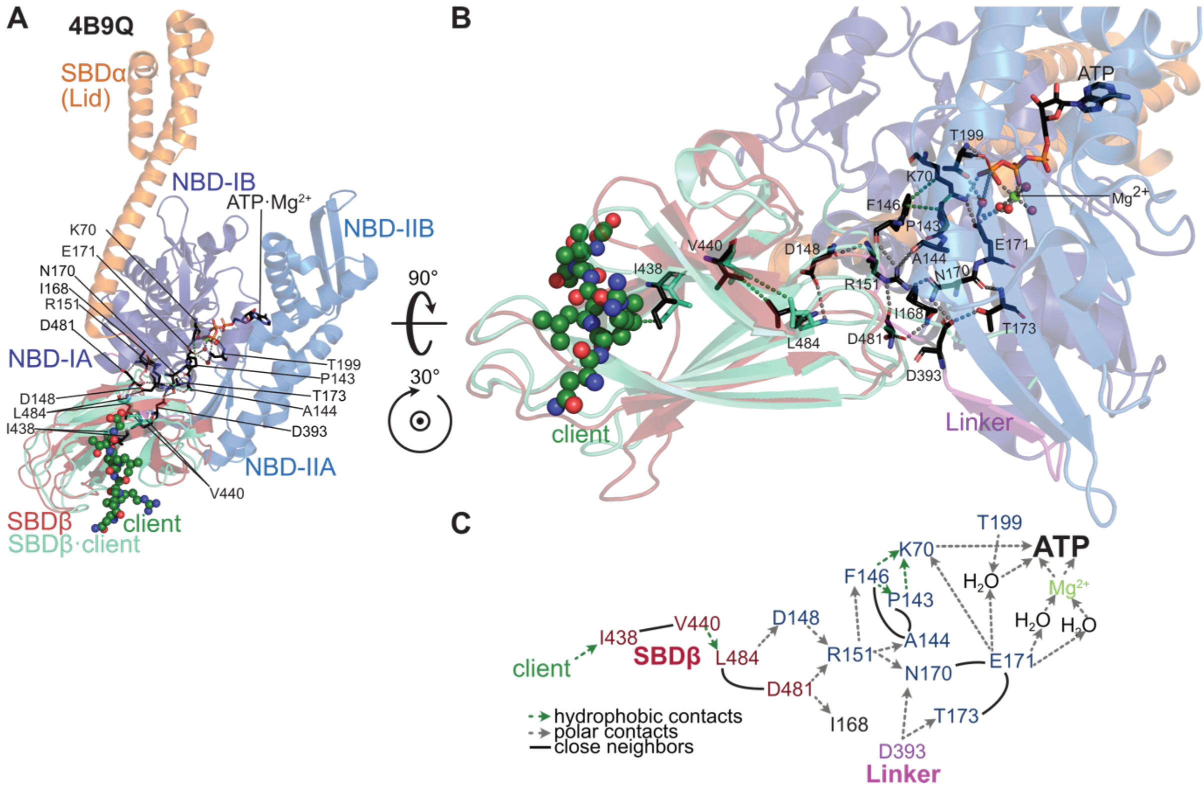

Figure S2. Allosteric signaling pathway for protein-induced conformational changes in DnaK·ATP.

A) Overview over the protein client allosteric signaling pathway. Shown is the ATP-bound structure (PDB ID 4B9Q) of DnaK (colored as in Fig. S1) that is thought to be most similar to the protein client and ATP bound state of Hsp70 with an overlay of the peptide client bound isolated SBDβ (PDB ID 1DKX; in light green and client peptide in atom colors with carbon in dark green). Depicted are residues that have been demonstrated to be important to process protein client-related allosteric signals in Hsp70 chaperones and are crucial to allow clients to stimulate the hydrolysis of ATP by Hsp70s; residues are shown as sticks in atom colors with carbon in black (NBD), dark read (4B9Q SBDβ), or light green (1DKX SBDβ). Note that, up to date, there is no structure of ATP-bound Hsp70 in complex with a protein client published and most of the residues in the isolated SBDβ overlay with the SBDβ in the ATP-bound state.

B) Zoom into the allosteric signaling pathway transmitting the client signal and residues that connect the client binding site with the ATP hydrolyzing active site rotated as compared to panel A as indicated.

C) Simplified overview over the protein client allosteric signaling pathway. Green dashed lines indicate hydrophobic interactions (C-C-distance, <4.2Å), gray dashed lines electrostatic interactions (O-O or N-O-distance <3.5Å), and black solid lines connection through the peptide backbone.

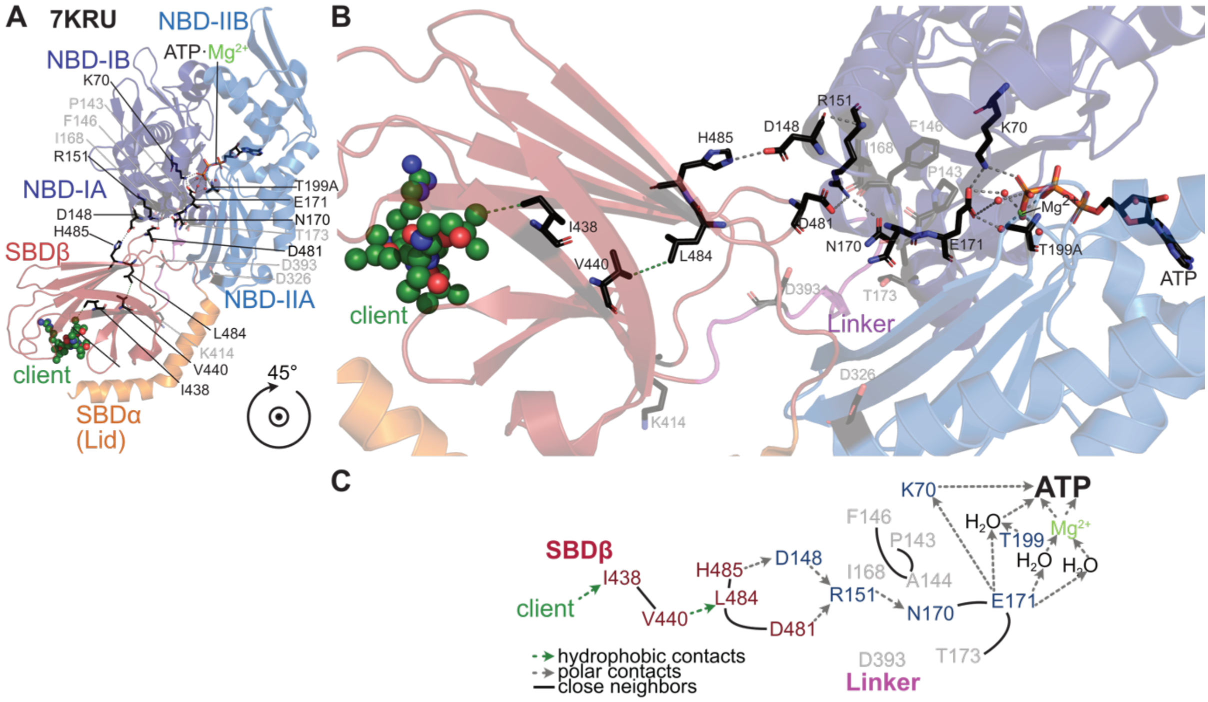

Figure S3. Allosteric signaling pathway for peptide-induced conformational changes in DnaK·ATP.

A) Overview over the peptide client allosteric signaling pathway. Shown is the peptide client and ATP-bound state of DnaK (PDB ID 7KRU) and residues that have been demonstrated to be important to process protein client-related allosteric signals in Hsp70 chaperones and are crucial to allow clients to stimulate the hydrolysis of ATP by Hsp70s. Based on our previously published data, showing that peptides and protein clients induce different reaction intermediates that stimulate the hydrolysis of ATP by Hsp70 to differing extents, we want to suggest that the allosteric signaling pathway for peptide clients is different from protein clients (1).

B) Zoom into the peptide client allosteric signaling pathway and residues that connect the client binding site with the active site for ATP hydrolysis. Residues that are important for allostery and that have lost their polar or hydrophobic interactions in this conformation are shown in gray.

C) Simplified overview over the peptide client allosteric signaling pathway. Green dashed lines indicate hydrophobic interactions (C-C-distance, <4.2Å), gray dashed lines electrostatic interactions (O-O or N-O-distance <3.5Å), and black solid lines connection through the peptide backbone.

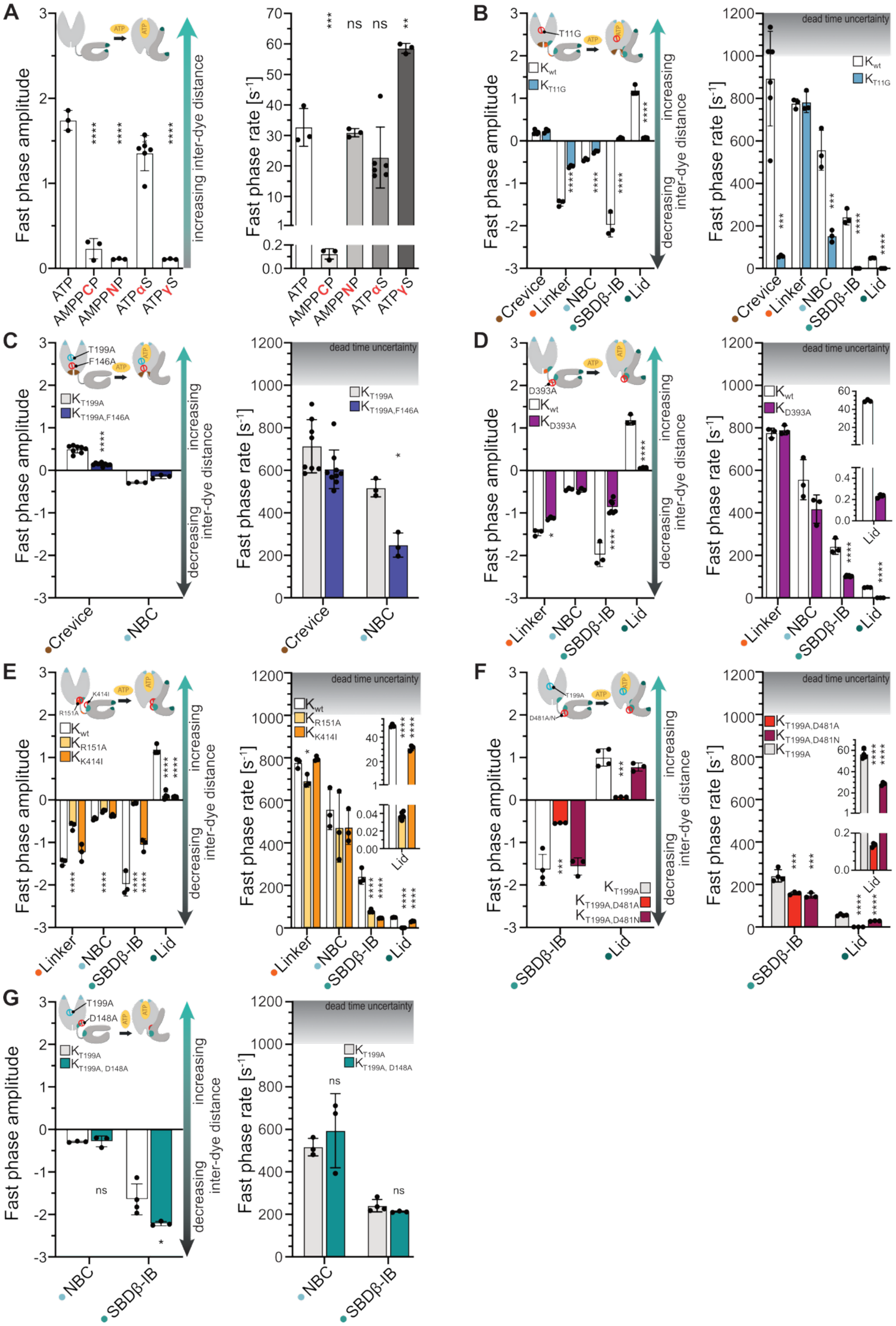

Figure S4. Comparison of the fast phase amplitudes and rates for DnaK$_{wt}$ and individual allosterically impaired DnaK variants.

A) Amplitudes and rates for binding of ATP and ATP analogues to the subpopulation of $DnaK_{lid}$ molecules constituting the fast phase of the reaction (related to Fig. 2B). Statistical significance of the values was assessed with ordinary one-way ANOVA and Dunnett's multiple comparison; ns, not significant; **, $p < 0.01$; ***, $p < 0.001$; ****, $p < 0.0001$.
B-G) Amplitudes and rates for ATP binding to the subpopulation of molecules constituting the fast phase of the reaction for $apoDnaK_{T11G}$ (**B**), $apoDnaK_{T199A,F146A}$ (**C**), $apoDnaK_{D393A}$ (**D**), $apoDnaK_{R151A}$ and $DnaK_{K414I}$ (**E**), $apoDnaK_{T199A,D481A}$ and $DnaK_{T199A,D481N}$ (**F**), and $apoDnaK_{T199A,D148A}$ (**G**). As reference for all experiments, amplitudes and rates of ATP-induced fluorescence changes in $DnaK_{wt}$ (B, D, E) or $DnaK_{T199A}$ (C, F, G) are shown. Note: the shaded area indicates the increased uncertainty of rates larger than 1,000 s^{-1} due to the dead time of the stopped-flow instrument. Statistical significance was assessed using t-tests for the crevice data and ordinary one-way ANOVA with Šídák's multiple comparison for all other comparisons (see explanation on statistical significance assessment in Experimental Procedures); *, $p<0.05$; **, $p < 0.01$; ***, $p < 0.001$; ****, $p < 0.0001$; no indications, differences are not significant.

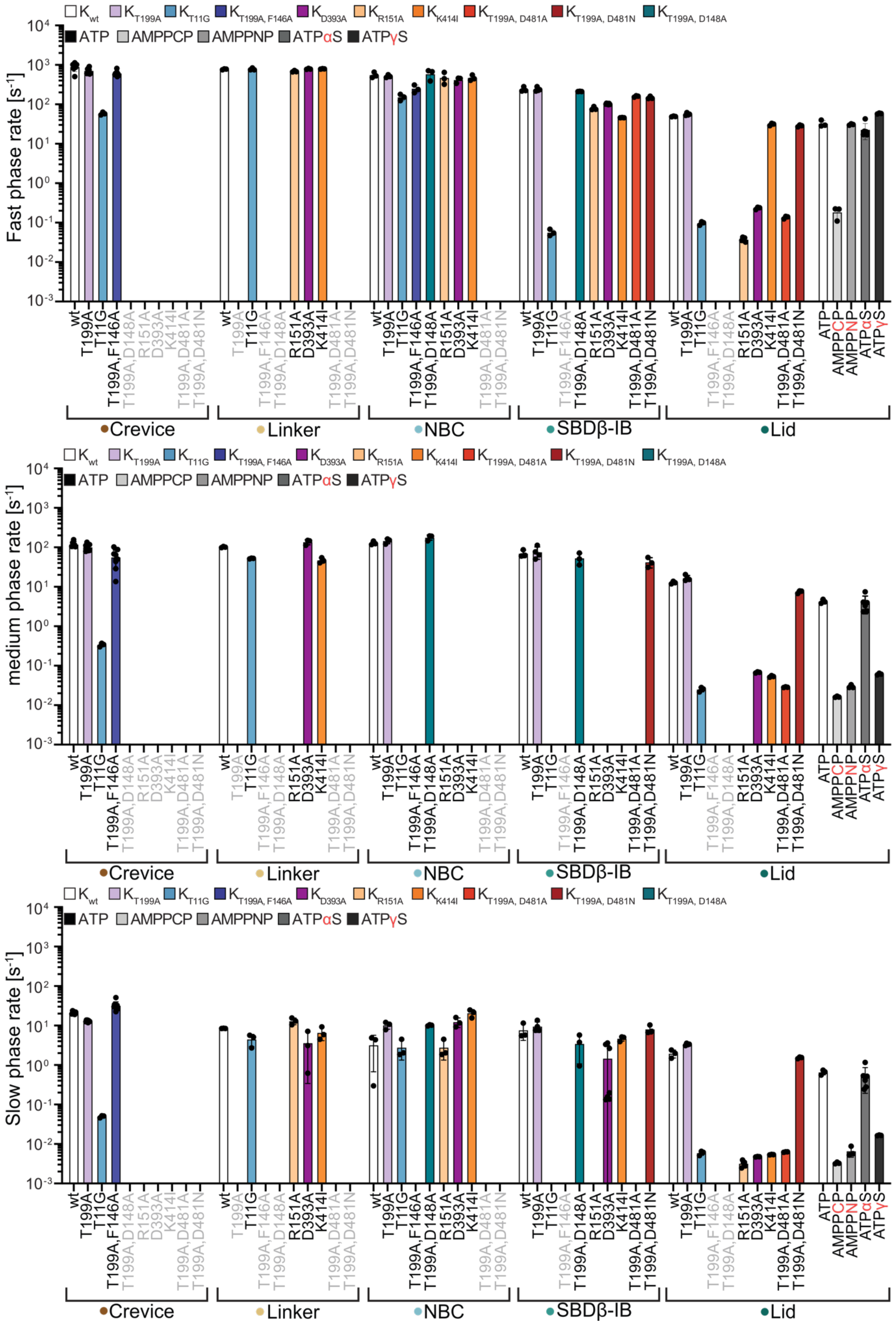
Fast phase rate [s-1]
medium phase rate [s-1]
Slow phase rate [s-1]
ATP AMPPCP AMPPNP ATPαS ATPγS
Crevice
Linker
NBC
SBDβ-IB
Lid

Figure S5. Rates for the individual conformational changes used to calculate weighted rates.

Rates for the fast (top), medium (middle), and slow (bottom) phases of the ATP-induced conformational changes in the double cysteine DnaK probes used to calculate the weighted rates that are shown in the figures of the main body of this study. For DnaK-variants depicted in gray the respective conformational change was not determined. Weighted rates were calculated with the equation $k_w = \sum_{i=1}^{3} f_i \cdot k_i$ where f_i is the relative contribution of an individual phase to the total amplitude and k_i is the rate constant of the individual phase. Shown are the mean and standard deviation for at least three replicates.

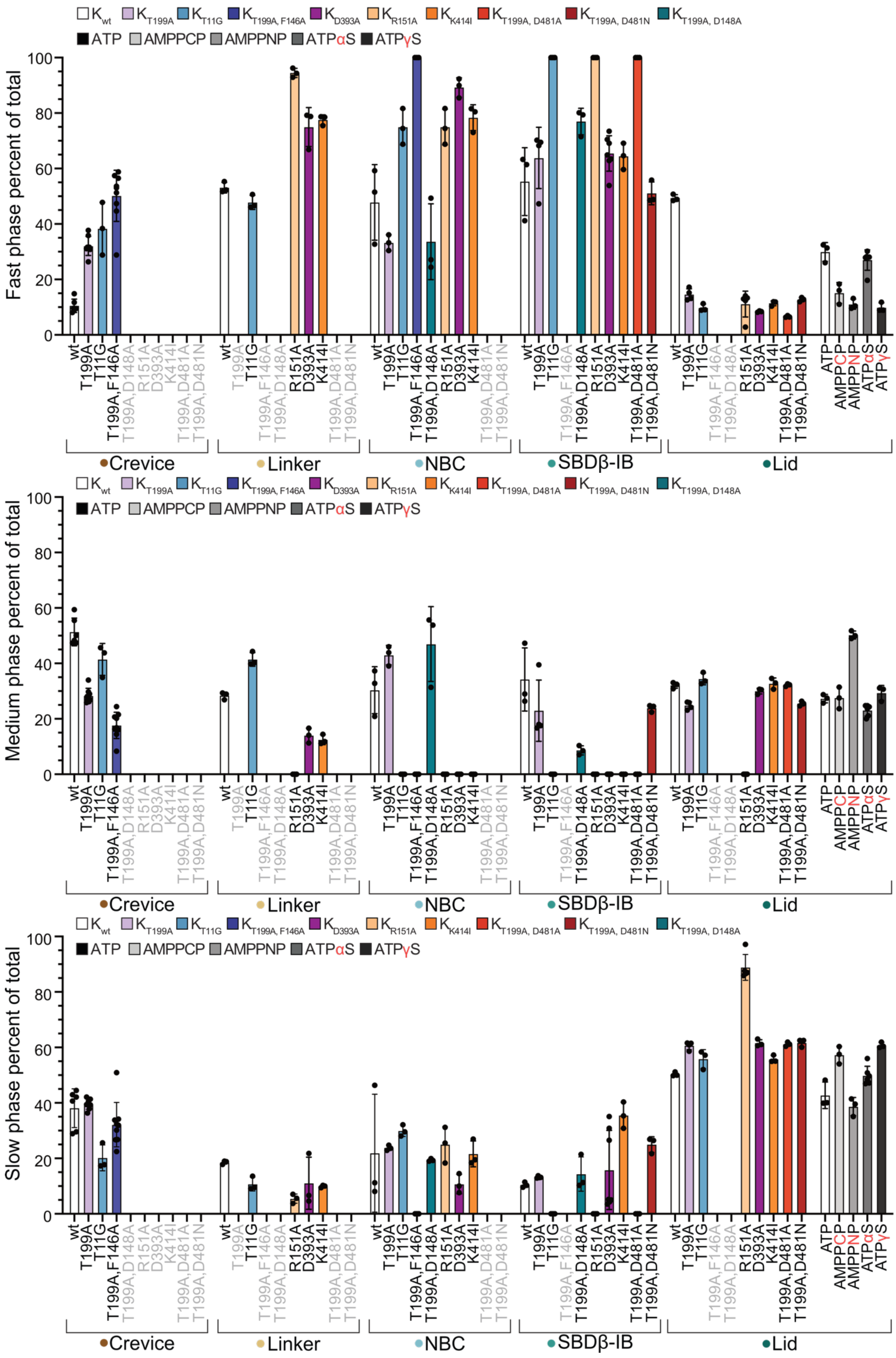
K wt
K T199A
K T11G
K T199A, F146A
K D393A
K R151A
K K414I
K T199A, D481A
K T199A, D481N
K T199A, D148A
ATP
AMPPCP
AMPPNP
ATPαS
ATPγS
Fast phase percent of total
Medium phase percent of total
Slow phase percent of total
Crevice
Linker
NBC
SBDβ-IB
Lid

Figure S6. Contribution of individual phases to the total amplitude for measured conformational changes used to calculate weighted rates.

Contribution of the fast (top), medium (middle), and slow (bottom) phases to the total amplitude for the individual phases of the ATP-induced conformational changes in the double cysteine DnaK probes used to calculate the weighted rates that are depicted in the figures of the main body of this study. Shown are the mean and standard deviation for at least three replicates.

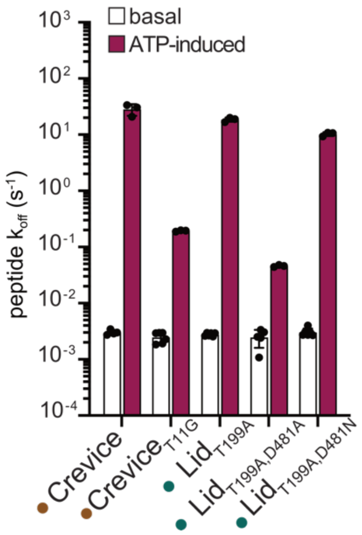

Figure S7. Client release rates in the presence and absence of ATP for selected DnaK variants.

Client release rates from DnaK in the presence or absence of ATP for selected DnaK double cysteine probes (crevice, $DnaK_{T22C,E213C}$; lid, $DnaK_{E430C,R547C}$) in absence and presence of amino acid replacements impacting allostery. Note, labeled protein cannot be used in this assay because the signal from the fluorescent dyes interferes with the signal obtained for the fluorescently labeled peptide. Shown are the mean and standard deviation for at least three replicates.

Movie S1. ATP-coordination and allosteric signaling pathways in the DnaK·ATP conformation

Cartoon representation of the ATP-bound, domain-docked conformation of DnaK (4B9Q) with NBD subdomain IA in dark blue, NBD subdomain IB in cyan, NBD subdomain IIA in marine blue, NBD subdomain IIB in deep teal, linker in purple, SBDβ in dark red, and SBDα in orange. Residues coordinating ATP and important for allostery are shown as sticks in atom colors with carbon in the color of the respective subdomain. Green dashed lines indicate hydrophobic interactions (C-C-distance, <4.2Å), gray dashed lines electrostatic interactions (O-O or N-O-distance <3.5Å) between backbone amides and ATP and between residues, and dark red dashed lines electrostatic interactions (O-O or N-O-distance <3.5Å) between sidechain functional groups and ATP. Of note, $DnaK_{T199A}$ was crystallized, therefore T199 is labeled as A199.

Movie S2. ATP-coordination and allosteric signaling pathways in the DnaK·ATP·peptide conformation

Cartoon representation of the ATP- and peptide-bound conformation of DnaK (7KRU/7KRW) with NBD subdomain IA in dark blue, NBD subdomain IB in cyan, NBD subdomain IIA in marine blue, NBD subdomain IIB in deep teal, linker in purple, SBDβ in dark red, and SBDα

in orange. Residues coordinating ATP and important for allostery are shown as sticks in atom colors with carbon in the color of the respective subdomain. Green dashed lines indicate hydrophobic interactions (C-C-distance, <4.2Å), gray dashed lines electrostatic interactions (O-O or N-O-distance <3.5Å) between backbone amides and ATP and between residues, dark red dashed lines electrostatic interactions (O-O or N-O-distance <3.5Å) between sidechain functional groups and ATP, and pale cyan dashed lines connect atoms that from electrostatic interactions in the ATP-bound, domain-docked conformation of DnaK (4B9Q) but are beyond a distance for electrostatic interactions in the ATP- and peptide-bound conformation of DnaK (7KRU/7KRW). Of note, $DnaK_{T199A}$ was crystallized, therefore T199 is labeled as A199.

References for SI

1. Rohland, L., Kityk, R., Smalinskaite, L., and Mayer, M. P. (2022) Conformational dynamics of the Hsp70 chaperone throughout key steps of its ATPase cycle Proc Natl Acad Sci U S A **119**, e2123238119 10.1073/pnas.2123238119
2. Sousa, M. C., and McKay, D. B. (1998) The hydroxyl of threonine 13 of the bovine 70-kDa heat shock cognate protein is essential for transducing the ATP-induced conformational change. Biochemistry **37**, 15392-15399 10.1021/bi981510x
3. O'Brien, M. C., and McKay, D. B. (1993) Threonine 204 of the chaperone protein Hsc70 influences the structure of the active site, but is not essential for ATP hydrolysis The Journal of biological chemistry **268**, 24323-24329,
4. Vogel, M., Bukau, B., and Mayer, M. P. (2006) Allosteric regulation of Hsp70 chaperones by a proline switch. Molecular Cell **21**, 359-367 10.1016/j.molcel.2005.12.017
5. Vogel, M., Mayer, M. P., and Bukau, B. (2006) Allosteric regulation of Hsp70 chaperones involves a conserved interdomain linker. The Journal of biological chemistry **281**, 38705-38711 10.1074/jbc.M609020200
6. Kityk, R., Vogel, M., Schlecht, R., Bukau, B., and Mayer, M. P. (2015) Pathways of allosteric regulation in Hsp70 chaperones. Nature communications **6**, 8308 10.1038/ncomms9308
7. Theyssen, H., Schuster, H. P., Packschies, L., Bukau, B., and Reinstein, J. (1996) The second step of ATP binding to DnaK induces peptide release. Journal of Molecular Biology **263**, 657-670 10.1006/jmbi.1996.0606
8. Wang, W., Liu, Q., Liu, Q., and Hendrickson, W. A. (2021) Conformational equilibria in allosteric control of Hsp70 chaperones Mol Cell **81**, 3919-3933 e3917 10.1016/j.molcel.2021.07.039
9. Bertelsen, E. B., Chang, L., Gestwicki, J. E., and Zuiderweg, E. R. P. (2009) Solution conformation of wild-type E. coli Hsp70 (DnaK) chaperone complexed with ADP and substrate. Proceedings of the National Academy of Sciences **106**, 8471-8476 10.1073/pnas.0903503106
10. Kityk, R., Kopp, J., Sinning, I., and Mayer, M. P. (2012) Structure and dynamics of the ATP-bound open conformation of Hsp70 chaperones. Molecular Cell **48**, 863-874 10.1016/j.molcel.2012.09.023
11. Wei, J., Gaut, J. R., and Hendershot, L. M. (1995) In vitro dissociation of BiP-peptide complexes requires a conformational change in BiP after ATP binding but does not require ATP hydrolysis The Journal of biological chemistry **270**, 26677-26682,

12. Barthel, T. K., Zhang, J., and Walker, G. C. (2001) ATPase-defective derivatives of Escherichia coli DnaK that behave differently with respect to ATP-induced conformational change and peptide release Journal of Bacteriology **183**, 5482-5490 10.1128/JB.183.19.5482-5490.2001
13. Montgomery, D. L., Morimoto, R. I., and Gierasch, L. M. (1999) Mutations in the substrate binding domain of the Escherichia coli 70 kDa molecular chaperone, DnaK, which alter substrate affinity or interdomain coupling. Journal of Molecular Biology **286**, 915-932 10.1006/jmbi.1998.2514
